# A single dorsal vagal complex circuit mediates the aversive and anorectic responses to GLP1R agonists

**DOI:** 10.1172/JCI193743

**Published:** 2026-09-15

**Authors:** Warren T. Yacawych, Yi Wang, Guoxiang Zhou, Shad Hassan, Cagri Bodur, Elisabeth Walters, John G. Santinga, Frederike Sass, Martin deVaux, Stace Kernodle, Iris Wu, Jenny M. Brown, Dylan Belmont-Rausch, Alan C. Rupp, Abigail J. Tomlinson, Zitian Lin, Emma VanTongeren, Anna Secher, Kirsten Raun, Tune H. Pers, Randy J. Seeley, Martin G. Myers, Weiwei Qiu

**Affiliations:** 1 Department of Internal Medicine, and; 2 Department of Molecular and Integrative Physiology, University of Michigan, Ann Arbor, Michigan, USA.; 3 Department of Metabolism and Endocrinology, National Clinical Research Center for Metabolic Diseases, The Second Xiangya Hospital, Central South University, Changsha, China.; 4 Zhejiang University-University of Edinburgh Institute, International Campus, Zhejiang University, Haining, China.; 5 Novo Nordisk Foundation Center for Basic Metabolic Research, University of Copenhagen, Copenhagen, Denmark.; 6 Center for Adipocyte Signaling (ADIPOSIGN), University of Southern Denmark, Odense, Denmark.; 7 Department of Surgery, University of Michigan, Ann Arbor, Michigan, USA.; 8 Global Drug Discovery, Novo Nordisk A/S, Maløv, Denmark.; 9 Research and Early Development, Novo Nordisk A/S, Bagsværd, Denmark.; 10 Department of Endocrinology, The Second Affiliated Hospital, School of Medicine, Zhejiang University, Hangzhou, China.; 11 International Joint Center for Biomedical Sciences (Haining), Zhejiang University, Haining, Zhejiang, China.; 12 ZJU-YST Joint Research Center for Fundamental Science, Zhejiang University, Hangzhou, Zhejiang, China.

**Keywords:** Metabolism, Neuroscience, Obesity

## Abstract

Glucagon-like peptide 1 (GLP-1) receptor agonists (GLP1RAs) effectively reduce feeding to treat obesity, although nausea and other aversive side effects of these drugs can limit their use. Brainstem circuits that promote satiation and mediate the physiological control of body weight can be distinguished from those that cause aversion. It remains unclear, however, whether brainstem *Glp1r* neurons contribute to the normal regulation of energy balance and whether GLP1RAs control appetite via circuits distinct from those that mediate aversive responses. Here, we silenced *Glp1r* neurons in the nucleus of the solitary tract (NTS) or area postrema (AP) (NTS^Glp1r^ or AP^Glp1r^ neurons, respectively) or restored their GLP1R signaling on an otherwise GLP1R-deficient background to determine physiological and pharmacological roles for each neuron population. Although NTS^Glp1r^ neurons contributed to the normal restraint of food intake and body weight, they failed to mediate GLP1RA-dependent weight loss. In contrast, while we detected no role for AP^Glp1r^ neurons in physiological feeding, they mediated both the weight-lowering and aversive effects of GLP1RAs. Therefore, while nonaversive NTS^Glp1r^ neurons controlled physiologic satiation they did not contribute to weight loss during GLP1RA treatment. Rather, AP^Glp1r^ neurons mediated both the weight-lowering and aversive effects of GLP1RAs, preventing the separation of their nauseating and weight-loss effects at a circuit level.

## Abstract 2

GLP-1 receptor agonists (GLP1RAs) cause nausea as well as anorexia. Although some neural systems suppress feeding withour nausea, GLP1RAs mediate both through a single system.

## Introduction

Obesity and its complications, including type 2 diabetes, represent enormous challenges to personal well-being and public health. While lifestyle changes such as exercise and/or dieting generally fail to mediate consistent and prolonged effects on body weight (1), Glucagon-like peptide 1 (GLP-1) receptor (GLP1R) agonists (GLP-1RAs) can produce sustained weight loss and improve cardiometabolic health for many people with obesity (2, 3).

Unfortunately, current GLP1RAs (including exenatide, liraglutide [Lira], semaglutide [Sema], and the dual GIP/GLP1R agonist tirzepatide [TZP]) promote nausea and other aversive gastrointestinal side effects in nearly two-thirds of patients taking these medications (2, 3), and many patients discontinue therapy (4). Thus, separating appetite suppression from the nauseating effects of GLP1-RAs could improve the treatment of obesity.

Recent evidence suggests that brainstem neuron populations responsible for promoting the aversive, nausea-associated responses to gut malaise are distinct from those that mediate the satiating responses to feeding (5–8). Indeed, while activating *Glp1r* neurons either in the area postrema (AP) (AP^Glp1r^ cells) or the nucleus tractus solitarius (NTS) (NTS^Glp1r^ cells) can dramatically reduce food intake, activating AP^Glp1r^ neurons (but not NTS^Glp1r^ neurons) promotes conditioned taste avoidance (CTA) (a surrogate for nausea in rodents) (6, 7). Similarly, while simultaneous ablation of AP^Glp1r^ and NTS^Glp1r^ neurons abrogates the suppression of food intake and body weight by GLP-1RAs, inhibition of AP^Glp1r^ neurons suffices to block CTA formation in response to GLP1RAs (6, 7). Hence, while only AP^Glp1r^ neurons can provoke (and are required to mediate) aversive responses, AP^Glp1r^ and NTS^Glp1r^ neurons can each suppress feeding.

While these results suggest that specifically targeting NTS^Glp1r^ neurons with GLP1RAs might promote satiation and weight loss in a manner that bypasses AP-mediated aversive effects, the extent to which using genetically engineered receptors to activate *Glp1r*-expressing neurons mimics long-term responses to GLP1RAs remains unclear, as do the individual contributions of AP^Glp1r^ and NTS^Glp1r^ neurons to GLP1RA-mediated weight loss. Furthermore, the roles for AP^Glp1r^ and NTS^Glp1r^ neurons in the physiological control of food intake and body weight remain undefined. To resolve these issues, we sought to compare the physiological roles for NTS^Glp1r^ and AP^Glp1r^ neurons in the control of feeding and body weight and to assess the ability of each cell type to mediate weight loss in response to GLP1RAs.

## Results

### Distinct roles for NTS^Glp1r^ and AP^Glp1r^ neurons in the physiologic control of body weight and aversion.

As previously reported by others (6, 7), chemogenetic activation of either AP^Glp1r^ neurons or NTS^Glp1r^ neurons suffices to decrease food intake and body weight, but only AP^Glp1r^ neurons promote CTA formation (Supplemental Figure 1; supplemental material available online with this article; https://doi.org/10.1172/JCI193743DS1). To determine the requirement for AP^Glp1r^ or NTS^Glp1r^ neurons in the physiologic control of energy balance, we injected AAV-DIO-TetTox-GFP (or control virus) into the AP or NTS of *Glp1r^Cre^* mice to mediate the Cre-dependent expression of tetanus toxin (TetTox [TT]) in AP^Glp1r^ or NTS^Glp1r^ mice (AP^Glp1r-TT^ and NTS^Glp1r-TT^ mice, respectively), silencing these neurons by preventing their release of neurotransmitters (Figure 1, A and B).

Following surgery, we measured food intake and body weight in AP^Glp1r-TT^ (Figure 1, C and D) and NTS^Glp1r-TT^ (Figure 1, E and F) mice during a 5–6 weeks of a normal chow diet followed by another 6–7 weeks of high-fat diet (HFD) feeding. Silencing AP^Glp1r^ neurons failed to alter food intake or body weight compared with controls on either diet (Figure 1, C and D). In contrast, although silencing NTS^Glp1r^ neurons did not detectably alter food intake or body weight compared with controls in chow-fed NTS^Glp1r-TT^ mice, these animals consumed approximately 25% more food and gained almost twice as much weight as the control mice over the 6 weeks of HFD feeding (Figure 1, E and F). Altogether, these results suggest that NTS^Glp1r^ neurons, but not AP^Glp1r^ neurons, restrained physiological feeding and body weight during exposure to a HFD.

Because of the difficulty of efficiently transducing AP cells without viral leak into the adjacent NTS, we assessed the potential efficacy of AP^Glp1r^ neuron silencing in AP^Glp1r-TT^ mice by examining Lira-induced FOS immunoreactivity (IR) in the parabrachial nucleus (PBN) (a major downstream projection target of AP^Glp1r^ neurons) (Supplemental Figure 2). We found an approximately 70% decrease in Lira-induced PBN FOS IR in AP^Glp1r-TT^ mice compared with controls (Supplemental Figure 2), although the number of GLP1R IR neurons in the AP remained unchanged (Supplemental Figure 3). Thus, TetTox blocked neurotransmitter release from AP^Glp1r^ neurons sufficiently to blunt this downstream response to Lira, and inefficient viral transduction of AP^Glp1r^ neurons was unlikely to explain the unaltered energy balance phenotype of AP^Glp1r-TT^ mice.

Taken together with the results of previous circuit-based manipulations (6, 7), these results support the idea that distinct *Glp1r-*expressing DVC circuits control satiety (NTS^Glp1r^ neurons) versus sickness-related responses (AP^Glp1r^ neurons). Hence, it is tempting to speculate that GLP1RAs might cause aversion by activating AP^Glp1r^ neurons while promoting the nonaversive suppression of food intake and body weight via NTS^Glp1r^ neurons. We therefore sought to determine roles for AP^Glp1r^ or NTS^Glp1r^ in pharmacologic responses to GLP1RAs.

### AP GLP1R contributes to GLP1RA-mediated aversive and feeding responses.

To assess roles for AP GLP1R in the anorectic effects of Lira, we stereotactically injected AAV-Cre-mCherry into the AP of *Glp1r^fl/fl^* mice (generating AP^Glp1r-KO^ mice predicted to lack *Glp1r* in the AP); an injection of AAV-tdTomato generated control AP^Ctrl^ mice (Supplemental Figure 3, A and B). This approach reduced AP GLP1R IR by approximately 60% in AP^Glp1r-KO^ mice compared with AP^Ctrl^ mice or WT mice that were injected with AAV-Cre-mCherry (Supplemental Figure 3, C–F); these AP^Glp1r-KO^ mice exhibited blunted Lira-induced CTA formation (Supplemental Figure 3G). Hence, decreasing AP *Glp1r* interfered with the aversive response to Lira in AP^Glp1r^ ^KO^ mice.

AP^Glp1rKO^ mice displayed a slightly attenuated anorectic response to Lira compared with controls during the first 4 hours of the dark cycle, but this effect was no longer statistically significant after 24 hours (Supplemental Figure 3H). While these data demonstrate that AP GLP1R contributes to the acute aversive and anorectic responses to Lira, the persistence of substantial AP GLP1R in AP^Glp1rKO^ mice prevented this approach from unequivocally defining the role for AP GLP1R in the long-term response to GLP1RAs.

### Site-specific reactivation of Glp1r expression in the DVC.

The inability to completely silence or abrogate *Glp1r* expression in AP^Glp1r^ neurons prevented the definitive delineation of roles for these neurons in the response to GLP1RAs, prompting us to revise our approach to this issue. To isolate the contributions of AP and NTS GLP1R to GLP1RA action, we turned to the *Glp1r^LoxTB^* allele, in which a LoxP-flanked transcription blocking cassette lies upstream of the fourth exon of the endogenous *Glp1r* gene (Figure 2A) (9). Cre removes the transcription blocker from the *Glp1r^LoxTB^* allele, reactivating native *Glp1r* expression on an otherwise *Glp1r*-null background.

Although we have substantial experience with comprehensive transduction of the NTS without viral leakage into the nearby AP (5, 10, 11), it is difficult to efficiently transduce the AP without leakage into the NTS. We thus set out to identify a marker gene specific to AP^Glp1r^ neurons that we could use as a Cre driver to permit the AP-specific genetic reactivation of *Glp1r* expression from the *Glp1r^LoxTB^* allele. We crossed *Glp1r^Cre^* onto the *Rosa26^tm5.1(CAG-Sun1-sfGFP)Nat^* background to promote the Cre-mediated expression of the nuclear envelope–labeling GFP-SUN1 fusion protein in *Glp1r* neurons (9, 12). We dissected the hypothalamus and DVC from these mice, used FACS sorting to enrich for GFP-containing nuclei, and subjected the resultant nuclei to single-nucleus RNA-seq (snRNA-seq) (Supplemental Figure 4A).

This process identified 43,919 *Glp1r* nuclei, which we separated into DVC and mediobasal hypothalamic (MBH) fractions by integrating these results with previous snRNA-seq datasets for each region (13) (Supplemental Figure 4A). The former 2 datasets were generated in the same experimental batches, allowing us to map the *Glp1r* nuclei with high confidence to either the hypothalamic or DVC cell populations. We then leveraged a separate, high-resolution snRNA-seq dataset comprising both DVC and MBH cell populations along with rat spatial transcriptomics data to identify AP *Glp1r* cell populations (14) (Supplemental Figure 4, B and C).

By systematically identifying genes distinguishing AP^Glp1r^ cells from non-AP *Glp1r* cell populations, we identified *Pirt* as the most commonly expressed AP^Glp1r^ marker gene (Supplemental Figure 4D). Examining *Pirt* expression across all DVC clusters revealed that *Pirt* was highly expressed in the AP (Supplemental Figure 4E), including in AP^Glp1r^ cell populations (Supplemental Figure 4F). We observed minimal *Pirt* expression in *Glp1r-*expressing nuclei outside of the AP, however. Unfortunately, we found no similarly selective marker genes for NTS^Glp1r^ neurons using similar methods.

We crossed the previously described *Pirt^Cre^* allele (15) onto the *Glp1r^LoxTB^* background to generate *Pirt^Cre^ Glp1r^LoxTB/LoxTB^* mice predicted to express *Glp1r* specifically in the AP (AP^Glp1r-Re^ mice; Figure 2A). We also injected AAV-Cre-GFP into the NTS of *Glp1r^LoxTB/LoxTB^* mice to restore *Glp1r* expression in NTS^Glp1r^ neurons (NTS^Glp1r-Re^ mice) (Figure 2A). As expected, ISH detected no *Glp1r* in *Glp1r^LoxTB/LoxTB^* (Glp1r^KO^) mice, *Glp1r* was restricted to the AP in the DVC of AP^Glp1r-Re^ mice, and only a few other *Glp1r*-expressing neurons were detected elsewhere (in the paraventricular thalamus [PVT]) in these animals (Figure 2, B and C, and Supplemental Figure 6). While *Pirt* is expressed in some areas of the PNS (15), which we did not examine exhaustively, *Glp1r* outside the brain does not meaningfully contribute to the weight-reducing effects of GLP1RAs (16–18). NTS^Glp1r-Re^ mice showed NTS *Glp1r* expression levels similar to levels seen in Glp1r^WT^ controls — although we also detected small numbers of *Glp1r*-expressing neurons in the AP of these animals (Figure 2, B and D, and Supplemental Figure 5, A and B), suggesting the uptake of a few AAV-Cre-GFP genomes into small numbers of terminals (19–21) from NTS-projecting AP^Glp1r^ neurons.

Examination of GLP1R IR in all genotypes revealed results similar to those with ISH (Supplemental Figure 5, C–F). We also found that AP^Glp1r-Re^ animals exhibited Lira-dependent CTA formation and FOS accumulation in calcitonin gene related peptide (CGRP) neurons of the PBN (Supplemental Figure 5, G–I). Thus, *Pirt^Cre^* specifically and robustly restored AP *Glp1r* expression and GLP1R signaling. NTS^Glp1r-Re^ mice exhibited *Glp1r* restoration in the NTS but also showed *Glp1r* restoration in a small number of AP^Glp1r^ neurons.

To define roles for *Glp1r* in the AP and NTS for the effects of acute Lira treatment, we administered Lira or vehicle to Glp1r^WT^, Glp1r^KO^, AP^Glp1r-Re^, and NTS^Glp1r-Re^ mice at the onset of the dark cycle and measured food intake over the subsequent 4 hours (Figure 2E). Although Lira failed to significantly decrease food intake in Glp1r^KO^ mice, it suppressed food intake in Glp1r^WT^ animals by approximately 80% during the first 4 hours of the dark cycle. Lira also suppressed food intake in NTS^Glp1r-Re^ and AP^Glp1-Re^ animals, although the suppression of food intake in NTS^Glp1r-Re^ mice (~35%) was less pronounced than in AP^Glp1r-Re^ mice (~60%). Furthermore, Lira decreased the uptake of gavaged paracetamol into the bloodstream (a measure of the gastric emptying rate) in Glp1r^WT^, NTS^Glp1r-Re^, and AP^Glp1r-Re^ mice (Figure 2F).

### AP GLP1R mediates the GLP1RA-dependent long-term suppression of food intake and body weight.

These data suggest that GLP1RAs can act via endogenous GLP1R in the AP and in the NTS to acutely suppress feeding at the onset of the dark cycle and to suppress gastric emptying. While other *Glp1r* neurons (e.g., in the hypothalamus and nodose ganglia) can mediate acute responses (defined as those observable in the hours immediately following a single dose) to GLP1RAs, these effects wane by 24 hours, and chronic GLP1R action on these cells does not change body weight (16, 17). Therefore, we sought to determine whether AP^Glp1r^ and NTS^Glp1r^ neurons suffice to mediate the long-term anorectic effects of GLP1RAs (Figures 3, 4, 5)

We initially examined long-term responses to Lira in weight-matched, chow-fed Glp1r^WT^, Glp1r^KO^, NTS^Glp1r-Re^, and AP^Glp1r-Re^ animals (Figure 3 and Supplemental Figure 7). We treated animals with vehicle for 2 days, Lira for 7 days, and then with vehicle for an additional 2 days. We monitored their food intake and body weight each day at the time of treatment, 1 hour prior to the onset of the dark cycle (Figure 3, A and B). Lira decreased food intake by approximately 40% in lean Glp1r^WT^ animals over the first day of treatment, although food intake in these animals subsequently returned toward baseline levels despite ongoing treatment (Figure 3A). Similarly, Lira decreased body weights of Glp1r^WT^ mice for the first 3 days of treatment only, after which body weights began to increase toward baseline (Figure 3B).

Although Lira provoked no changes in food intake or body weight in Glp1r^KO^ mice, the suppression of food intake in AP^Glp1r-Re^ mice mirrored that of Glp1r^WT^ animals (Figure 3, A and B). Interestingly, we detected no alterations in food intake or body weight in NTS^Glp1r-Re^ animals. These findings suggest that AP GLP1R suffices to mediate the majority of long-term GLP1RA action on feeding and body weight but that NTS GLP1R mediates little, if any, long-term response to GLP1RAs.

Given the strong suppression of feeding and body weight in response to the chemogenetic activation of NTS^Glp1r^ neurons and the important role that NTS^Glp1r^ neurons play in the physiologic control of energy balance (7) (Figure 1 and Supplemental Figure 1), the fact that NTS^Glp1r-Re^ animals did not respond to GLP1RAs was somewhat surprising. Chow-fed mice exhibit relatively modest responses to Lira that can attenuate over time (17), presumably due to the strength of their counter-regulatory responses to weight loss, including low leptin. Hence, we decided to examine the responses of obese Glp1r^WT^, Glp1r^KO^, NTS^Glp1r-Re^, and AP^Glp1r-Re^ animals to GLP1RAs.

Following several weeks of HFD feeding, when all groups had similarly elevated body weights (Supplemental Figure 7B), we reassessed the animals’ response to Lira during HFD feeding (Figure 3, C–F). As predicted, Lira promoted a sustained decrease in food intake and body weight in obese Glp1r^WT^ mice (Figure 3, C–F). Furthermore, Lira stimulated a similarly large and sustained suppression of food intake and body weight in AP^Glp1r-Re^ animals. However, Lira once again failed to alter food intake and body weight in HFD-fed NTS^Glp1r-Re^ and Glp1r^KO^ animals. Thus, restoring AP *Glp1r* expression alone produced a Lira response similar to that of Glp1r^WT^ mice, while reactivating *Glp1r* expression in the NTS failed to restore any long-term Lira responses. These data suggest that NTS GLP1R contributed negligibly to the weight-lowering effects of Lira, while GLP1R in AP^Glp1r^ neurons sufficed to mediate most long-term Lira-induced suppression of feeding and body weight.

While Lira durably reduces feeding and body weight, Sema and TZP provoke stronger weight-lowering effects in rodents and humans (3). The ability of TZP to activate the glucose-dependent insulinotropic polypeptide (GIP) receptor (GIPR) in addition to the GLP1R probably contributes to its efficacy in humans, although it activates the murine GIPR less potently than the human receptor (22, 23). Proposed mechanisms for the increased weight loss efficacy of Sema compared with Lira include its longer half-life and improved ability to access brain regions behind the blood brain barrier (BBB) (24), like the NTS (25). To determine whether the properties of Sema and TZP might enable NTS^Glp1r^ cells to mediate GLP1RA-stimulated weight loss, we examined the responses to these compounds in obese Glp1r^WT^, Glp1r^KO^, NTS^Glp1r-Re^, and AP^Glp1r-Re^ animals (Figure 4).

We examined the response to Sema in the same cohort of animals used for the Lira studies but generated a new cohort of experimental animals to test the response to TZP. Because others have reported that endogenous *Glp1r* expression affects energy balance in HFD-fed mice (17, 26), we measured the body weights of Glp1r^WT^, Glp1r^KO^, NTS^Glp1r-Re^, and AP^Glp1r-Re^ animals at the beginning of each treatment period and assessed weight gain over the initial period on HFD feeding (Supplemental Figure 7). We noted that the body weights of NTS^Glp1r-Re^ tended to be higher than those of Glp1r^WT^ and AP^Glp1r-Re^ animals and that NTS^Glp1r-Re^ animals also gained more weight than these other lines during the initial period on a HFD. We also noted that Glp1r^KO^ animals tended to be heavier than Glp1r^WT^ and AP^Glp1r-Re^ animals. Thus, the lack of *Glp1r* in the *Glp1r^LSL/LSL^* model did not protect against obesity. The trends in our data are consistent either with the presence of AP *Glp1r* being protective relative to the complete absence of *Glp1r* and/or *Glp1r* in the NTS being obesogenic.

We extended the duration of treatment with Sema and TZP compared with Lira to enhance our ability to detect any potentially small effects of GLP1RAs in the NTS^Glp1r-Re^ animals. As with Lira, we found that Sema (Figure 4, A–D) and TZP (Figure 4, E–G) promoted long-term decreases in food intake and body weight in obese AP^Glp1r-Re^ and Glp1r^WT^ mice. Once again, however, treatment with either of these GLP1RAs failed to decrease food intake and body weight in NTS^Glp1r-Re^ animals relative to Glp1r^KO^ controls.

Interestingly, while Sema decreased body weight similarly in AP^Glp1r-Re^ and Glp1r^WT^ mice (Figure 4, A–D), TZP decreased body weight to a lesser degree in AP^Glp1r-Re^ mice relative to controls (Figure 4, E–H). This effect was driven by the more limited response of female AP^Glp1r-Re^ mice, which lost approximately 50% less weight on TZP than did their male counterparts (Supplemental Figure 8). While this phenomenon was not driven by different beginning body weights (Supplemental Figure 7), we noted some variability in the extent of AP *Glp1r* expression in AP^Glp1r-Re^ mice (Figure 2D). Hence, we speculate that decreased *Pirt^Cre^*-mediated AP *Glp1r* reactivation in female AP^Glp1r-Re^ mice might have limited their maximal response to TZP. It is not clear why we observed this effect more prominently during treatment with TZP than with other GLP1RAs, however.

Overall, these results reveal that (a) endogenous GLP1R on AP^Glp1r^ neurons suffices to mediate the weight-lowering effects of each of the tested GLP1RAs; (b) endogenous GLP1R on NTS^Glp1r^ neurons minimally contributes to GLP1RA-dependent weight loss; and (c) the improved efficacy of TZP and Sema compared with Lira does not reflect any enhanced ability to act via endogenous GLP1Rs on NTS^Glp1r^ neurons in mice.

### Overexpressed NTS GLP1R fails to enhance the long-term suppression of food intake and body weight by GLP1RAs.

Given that the designer receptors exclusively activated by designer drugs–mediated (DREADD-mediated) activation of NTS^Glp1r^ neurons suffices to reduce feeding and body weight over several days (Supplemental Figure 1) (7), we were surprised by the inability of NTS^Glp1r^ neurons to mediate long-term GLP1RA action. *Glp1r* mRNA expression and GLP1R IR in the NTS were noticeably lower than in the AP, however (Figure 2, B and C, and Supplemental Figure 5, C–F). Thus, we reasoned that NTS^Glp1r^ neurons might contain insufficient GLP1R to mediate meaningful responses to GLP1RAs.

While excision of the LoxTB cassette from the *Glp1r^LoxTB^* allele (Figures 2–4) restored endogenous levels of *Glp1r* expression in Cre-containing cells, we sought to increase GLP1R in NTS^Glp1r^ neurons. We thus constructed AAV-hSyn-DIO-hGLP1R to mediate the Cre-dependent overexpression of human *GLP1R* (*hGLP1R*) and injected it (or control AAV-hSyn-DIO-Syn-mCherry) into the NTS of *Glp1r^Cre^* animals (NTS^hGLP1R-OX^ and Glp1r^WT^ mice, respectively) (Figure 5).

Immunostaining with a hGLP1R-selective antibody confirmed hGLP1R expression in the NTS but not elsewhere in the DVC of NTS^hGLP1R-OX^ mice (Figure 5, A and B). We initially tested the efficacy of Sema (which binds and activates both hGLP1R and murine GLP1R (mGLP1R)) in NTS^hGlp1r-OX^ mice (Figure 5, C, E–G, and K). Sema induced similar decreases in food intake and body weights in NTS^hGLP1R-OX^ mice and controls, however. Hence, either GLP1RA action on NTS^Glp1r^ neurons remained unable to alter energy balance despite *hGLP1R* overexpression or the magnitude of the AP^Glp1r^ neuron–mediated response to GLP1RAs prevented the detection of any effects resulting from *hGLP1R* overexpression in NTS^Glp1r^ neurons.

To distinguish between these possibilities, we examined the response of NTS^hGLP1R-OX^ mice and their controls to AZD0186, an oral GLP1RA specific for hGLP1R (27) (Figure 5, D, and H–O). We found that AZD0186 promoted FOS accumulation in the NTS of NTS^hGLP1R-OX^ mice but not controls (Figure 5, M–O), confirming the ability of hGLP1R in the NTS of NTS^hGLP1R-OX^ mice to respond acutely to this compound. The stress of gavage treatment decreased body weights of NTS^hGLP1R-OX^ mice and controls, but AZD0186 treatment of NTS^hGLP1R-OX^ mice for 7 days failed to further reduce food intake or body weight compared with control mice (Figure 5, D, and H–L). Therefore, neither endogenous mGLP1R nor overexpression of hGLP1R enabled NTS^Glp1r^ neurons to mediate the weight loss effects of GLP1RAs.

Collectively, these results underscore the central importance of AP^Glp1r^ neurons for the pharmacologic suppression of food intake and energy balance by currently available GLP1RAs. Because AP^Glp1r^ neurons mediate both the anorectic and aversive effects of GLP1RAs, these effects cannot be dissociated at a neuroanatomic level.

## Discussion

Here, we reveal distinct physiological and pharmacologic roles for brainstem *Glp1r–*expressing circuits in the control of feeding, body weight, and aversion. While nonaversive NTS^Glp1r^ neurons were required for the physiological long-term restraint of food intake and body weight, these cells failed to mediate weight loss in response to GLP1RAs. In contrast, AP^Glp1r^ neurons mediated both aversion and weight loss in response to GLP1RAs. Collectively, these results suggest that, despite the ability of chemogenetically activated AP^Glp1r^ or NTS neurons to suppress feeding (6, 7) (and our data here), (a) NTSGlp1r neurons fail to mediate weight loss in response to GLP1RAs, and (b) it is not possible to separate the aversive and appetite-suppressing effects of GLP1RAs.

### The AP as a target for GLP1RA-dependent weight loss.

While our data reveal the sufficiency of AP^Glp1r^ neurons for the weight loss effects of GLP1RAs, previous studies have found that lesioning the AP does not prevent Lira from reducing body weight (28, 29). Circumventricular organs (like the AP, median eminence, etc.) are responsible for gating access of circulating molecules across the BBB and into the surrounding brain tissue (30), however, and AP lesions cause hypophagia and weight loss, likely because of BBB damage (31, 32). Hence, although AP GLP1R mediates most GLP1RA-dependent weight loss in BBB-intact animals, it is possible that disruption of the BBB could enable sufficient GLP1RA access into the brain to stimulate additional GLP1R-containing circuits.

Some reports have also proposed roles for NTS^Glp1r^ neurons in the response to GLP1RAs in animals with an intact BBB: knocking down NTS *Glp1r* in rats slightly attenuates Lira’s ability to reduce body weight (28); this effect is modest relative to the effect of ablating all DVC (AP and NTS) *Glp1r* neurons (7), however, it is consistent with our finding that NTS^Glp1r^ neurons minimally contributed to weight loss by GLP1RAs relative to the contribution of AP^Glp1r^ cells.

Non-DVC *Glp1r*-expressing neurons, including those in the hypothalamus and vagus, have also been proposed to mediate GLP1RA-dependent weight loss. While GABAergic Glp1r neurons of the dorsomedial hypothalamic nucleus (DMHGlp1r) directly innervate and inhibit orexigenic arcuate nucleus (ARC) *Agrp* neurons (33), restoring DMH *Glp1r* permits the acute suppression of food intake by GLP1RAs, but not the long-term suppression of food intake and body weight (9, 34). Furthermore, deletion of *Glp1r* in all hypothalamic neurons or in the nodose ganglion, or ablation of *Glp1r*-expressing ARC neurons minimally affects weight loss responses to GLP1RAs (16–18).

Roles in GLP1RA-dependent feeding suppression have also been suggested for *Glp1r*-expressing neurons in the lateral septum (LS) (LS^Glp1r^ neurons) (35). Although LS^Glp1r^ neurons appear to restrain physiologic feeding (as do NTS^Glp1r^ neurons), ablation of *Glp1r* in the LS only slightly decreases the anorectic response to GLP1RAs (35). Consistently, the Sema-stimulated long-term transcriptional response in the LS (and NTS and DMH) pales by comparison to the response exhibited by the AP (13).

While our study identifies the AP as the major site of action for GLP1RA-induced weight loss, it remains possible that GLP1RA action via other *Glp1r-*expressing circuits modulates feeding-related processes that are poorly quantified by the measurement of daily food intake (e.g., reward, palatability, or post-ingestive feedback). It also remains possible that these other *Glp1r*-expressing circuits might modify AP^Glp1r^-mediated responses to GLP1RAs.

### Potential contributions of satiety to GLP1RA-stimulated weight loss.

Nausea can decrease feeding, and patients who report more nausea and vomiting tend to lose more weight during treatment with GLP1RAs, consistent with the dual role for AP^Glp1r^ neurons in aversion and weight loss (4, 22). However, some patients lose weight without side effects. Furthermore, many patients report that GLP1RAs cause increased satiety (the feeling of fullness that lasts until the next meal), suggesting a potential role for nonaversive circuits in GLP1RA-stimulated weight loss.

While activating all AP^Glp1r^ neurons promotes aversion, several lines of evidence suggest that AP^Glp1r^ neurons might comprise multiple functionally distinct subpopulations. Although the gut irritant LiCl increases intracellular Ca^2+^ (Ca^+2^_i_, a proxy for neuronal activity) in approximately half of all AP^Glp1r^ neurons in vivo, gastric nutrient infusion increases Ca^2+^_i_ in 41% of AP^Glp1r^ neurons, and these nutrient-activated AP^Glp1r^ neurons are distinct from those activated by LiCl (7). Furthermore, bioinformatics analyses suggest the existence of multiple transcriptionally distinct *Glp1r*-expressing AP neuron subpopulations (6, 36).

Because the AP^Glp1r^ neurons that sense gastrointestinal nutrients are distinct from those that respond to irritants, the nutrient-sensing subset of AP^Glp1r^ neurons could theoretically promote satiety. While HFD-fed AP^Glp1r-TT^ mice exhibited no increase in food intake or body weight, as might be expected for neurons that mediate physiologic satiation, we cannot rule out the possibility that incomplete silencing of AP^Glp1r^ neurons underlay this lack of effect.

AP^Glp1r^ neurons may recruit downstream satiety circuits in addition to aversive systems, however. Indeed, we previously showed that GLP1RAs can activate nonaversive, satiety-encoding NTS *Calcr* cells and that silencing these cells blunts short-term GLP1RA-dependent food intake suppression (5). Hence, although AP^Glp1r^ neurons (a) do not contribute to the physiological control of food intake or body weight and (b) promote major GLP1RA-dependent weight loss effects via pathways that mediate the aversive and nonphysiological suppression of food intake, some AP^Glp1r^ neurons can acutely activate satiety systems. It is possible that the recruitment of such satiety circuits is more robust or longer lasting in patients who report satiety responses to GLP1RA treatment.

### Failure of NTS^Glp1r^ neurons to mediate responses to GLP1RAs.

Because data from others and us show that chemogenetic activation of NTS^Glp1r^ neurons potently suppresses feeding and body weight (7), we were surprised by the failure of NTS^Glp1r^ neurons to mediate body weight reduction in response to GLP1RAs. Indeed, because the intra-NTS injection of AAV-Cre produced small numbers of GLP1R IR neurons in the AP of NTS^Glp1r-Re^ mice, we cannot exclude the possibility that these few *Glp1r*-expressing AP^Glp1r^ neurons mediated the modest acute effects of GLP1RAs observed in Glp1r^NTS-Re^ mice. Several limitations might account for the inability of NTS^Glp1r^ neurons to mediate GLP1RA-dependent weight loss.

First, the relatively small number of NTS^Glp1r^ neurons (especially compared with AP^Glp1r^ neurons) could limit the strength of the anorectic signal generated by this cell population. Second, while fenestrated capillaries provide AP neurons with direct access to circulating signals, the NTS resides behind the BBB, potentially limiting the access of circulating GLP1RAs to NTS^Glp1r^ neurons. Third, NTS^Glp1r^ neurons contain less detectable GLP1R than do AP^Glp1r^ neurons. Indeed, peripherally dosed fluorescent Lira and Sema accumulate in the NTS at lower levels than in the AP, and CNS concentrations of the hGLP1R-specific small molecular GLP1RA AZD0186 achieve only 4% of those in the circulation (24, 27). Hence, the small number of NTS^Glp1r^ neurons, their low GLP1R content, and the limited GLP1RA access to the NTS (or some combination of these factors) could prevent NTS^Glp1r^ neurons from responding adequately to circulating GLP1RAs.

However, the potent reduction in feeding and body weight produced by chemogenetically activating NTS^Glp1r^ cells, along with their crucial role in physiological body weight regulation, argues against the notion that the low number of NTS^Glp1r^ cells alone explains their inability to mediate GLP1RA responses. Furthermore, overexpression of *hGLP1R* in NTS^Glp1r^ neurons failed to augment the response to Sema (which activates both endogenous mGLP1R and overexpressed hGLP1R) or to enable weight loss responses to circulating AZD0186. Hence, low GLP1R by itself is unlikely to explain the inability of in NTS^Glp1r^ neurons to mediate weight loss responses to GLP1RAs.

Thus, the poor access of circulating GLP1RAs to the NTS may represent the main obstacle that prevents NTS^Glp1r^ neurons from mediating weight loss responses to these compounds. Indeed, as noted above, disruption of the BBB and increased GLP1RA access to areas such as the NTS might underlie the ability of GLP1RAs to provoke weight loss following AP lesions (28, 29). Hence, increasing GLP1RA access to the brain could enhance the ability of nonaversive *Glp1r* neuron populations (like NTS^Glp1r^ neurons) to mediate weight loss responses to GLP1RAs.

### Other effects of GLP1RAs.

While GLP1RAs were initially approved to treat diabetes, and then obesity, GLP1RAs may have beneficial effects beyond the control of blood glucose and body weight. GLP1RAs reduce the risk for cardiovascular events and renal dysfunction and can improve several forms of arthritis and autoimmune disease (3, 22). GLP1RAs promote antiinflammatory effects, which might contribute to these weight- and glucose-independent salutary responses to GLP1RAs. GLP1RAs have also demonstrated some efficacy in the treatment of substance use and addiction disorders. While our results demonstrate the importance of *Glp1r* in AP^Glp1r^ neurons for the aversive and long-term anorectic responses to GLP1RAs, it is not clear whether AP *Glp1r* mediates these other GLP1RA effects as well, or whether GLP1RAs may promote some or all of these effects by acting elsewhere in the brain or periphery.

## Methods

### Sex as a biological variable

The experiments in these studies utilized both male and female mice. Our study examined male and female animals, and some sex-dimorphic effects are reported.

### Animals

Mice for AP stereotaxic injections were bred and housed in animal colonies in the animal facility at Zhejiang University. All other animals were bred and housed in the Unit for Laboratory Animal Medicine at the University of Michigan.

Mice were housed in specific pathogen–free (SPF), temperature-controlled (22°C) rooms with 12-hour light/12-hour dark cycles. Mice were provided food (Purina Lab Diet 5LOD, unless otherwise specified) and water ad libitum (except as noted below), with daily health checks performed by qualified animal care personnel.

We purchased male and female C57BL/6 mice for experiments and breeding at the University of Michigan from The Jackson Laboratory. *Glp1r^Cre^* (JAX catalog 029283). *Glp1r^LSL^* (9), *Glp1r^fl^* (37), and *Pirt^Cre^* (15) animals have been previously described. *Glp1r^Cre^*, *Glp1r^fl^*, and *Glp1r^Stop-fl^* were propagated by intercrossing homozygous mice of the same genotype. We crossed *Pirt^Cre/+^ Glp1r^Stop-fl/Stop-fl^* animals with homozygous *Glp1r^Stop-fl/Stop-fl^* mice to generate *Pirt^Cre/+^ Glp1r^Stop-fl/Stop-fl^* (AP^Glp1r-Re^) and *Glp1r^Stop-fl/Stop-fl^* (Glp1r^KO^) animals for study.

For the AP stereotaxic injections, *Glp1r^Cre^* mice were purchased from the Shanghai Model Organisms Center (catalog NM-KI-200134); *Glp1r^fl^* mice were purchased from GemPharmatech (catalog T005818).

For all studies, animals were processed in order of the ear tag number, which was randomly assigned at the time of tailing (before genotyping). All mouse models (with the exception of purchased C57BL/6 mice) were on the segregating C57BL/6 SJL 129 genetic background; sibling controls were used throughout. Male and female mice were used for all experiments in which a single sex was not indicated.

### Viral reagents

AAV-Cre-GFP, AAV-GFP, AAV-DIO-hM3Dq-mCherry, and AAV-DIO-TetTox-GFP were as previously described (5) and were prepared as AAV8 serotype viruses by the University of Michigan Viral Vector Core. AAV-hSyn-DIO-hGlp1r was generated as described below and also packaged by the University of Michigan Viral Vector Core.

AAV-hSyn-tdTomato (S0266-8), AAV-hSyn-Cre-mCherry (AAV-Cre-mCherry, S0702-8), AAV-hSyn-DIO-hM3Dq-mCherry (S0192-8), and AAV-CAG-DIO-Tettox-EGFP (S0235-8) for AP injections were purchased from Shanghai Taitool Bioscience specifically for AP injections.

To generate AAV-hSyn-DIO-hGLP1R, we had Elegen synthesize double-floxed inverse orientation (DIO) human *GLP1R* transcript variant 1 mRNA (NM_002062) dsDNA sequence and subcloned this insert into pAAV-hSyn-DIO-EGFP plasmid (Addgene plasmid 50457) to replace the “DIO-EGFP” sequence for the construction of AAV helper plasmid pAAV-hSyn-DIO-hGLP1R. For subcloning, we used BamHI-HF (no. 3136), HindIII-HF (R3104), Instant Sticky-end Ligase Master Mix (no. M0370), and NEB 5-α Competent *E*. *coli* (high efficiency, no. C2987) from New England Biolabs. We performed restriction enzyme digestion, ligation, and transformation according to the manufacturer’s protocols. We had Eurofins Genomics confirm the sequence by Sanger sequencing, and the University of Michigan Medical School Vector Core produced rAAV8-hSyn-DIO-hGLP1R.

### Stereotaxic injections

For injection, following the induction of isoflurane anesthesia and placement in a stereotaxic frame, mice had their head rotated 90° downward for easy access to the neck. Neck muscles were carefully cut until the brainstem was exposed. For both AP and NTS surgeries, the obex was set as the reference point for injection. For NTS, the coordinates M/L = ± 0.3 mm (distance across fourth ventricle) to ± 0.25 mm, and D/V = –0.5 were used. The dura was then opened, and a guide cannula with a pipette injector was lowered into the NTS, and 50 nL virus was injected using a World Precision Instrument microinjector at a rate of 5–30 nL/minute. for the AP, the coordinates M/L = 0 mm and D/V = –0.2 mm were used. The dura were opened, and a guide cannula with a pipette injector was lowered into the AP and 30 nL adeno-associated viruses (AAVs) at a rate of 1 nL/s were injected using a brain stereotaxic injection apparatus (R480 glass microglass injection pump, RWD Life Science; Nanoject III, Drummond Scientific Company).

For AP and NTS surgeries, the injector was removed from the brain tissue 3 minutes after injection to allow for adequate dispersal and absorption of the virus. The incision site was closed and sutured. Mice received prophylactic analgesics before and after surgery (carprofen, 5 mg/kg, s.c.). All mice were at least 8 weeks of age at the time of injection.

Except for TetTox-expressing mice undergoing weekly longitudinal observational studies which were monitored immediately after surgery, all animals were allowed at least 3 weeks to recover following surgery before initiating the studies.

### Food intake and body weight studies

For acute food intake studies, animals on chow or a 60% HFD (Research Diets, D12492) were fasted 4 hours prior to the onset of the dark cycle. Immediately prior to the onset of the dark cycle, animals were given an i.p. injection of vehicle or Lira (400 μg/kg; Novo Nordisk). A known amount of food was then returned, and the remaining food was measured 2, 4, and 24 hours later.

For long-term food intake in hM3Dq-expressing mice, animals were administered saline i.p. for 1–2 days prior to injecting saline or clozapine-n-oxide (CNO) (C879476, Macklin or 1 mg/kg, 4936, Tocris Bioscience) (i.p., 1 mg/kg at 5 pm) followed by CNO (3.33 μg/mL) or vehicle given in the drinking water for the duration of treatment. Food intake and body weight were measured once daily at the onset of the dark cycle.

For chronic treatments with GLP-1RAs in the Lira studies (Figure 3), chow-fed or HFD-fed mice were administered vehicle (sodium phosphate 50 mM, sodium chloride 70 mM, 0.007% Tween 20, pH 8) for 2 days, followed by an increasing dosing paradigm over the first 3 days of 100 μg/kg, 300 μg/kg, and 500 μg/kg, after which the maximum dose was used throughout the treatment period.

For chronic Sema studies, mice were administered the aforementioned vehicle for 2 days, followed by an increasing dosing paradigm over the first 4 days of 2.5 nmol/kg, 5 nmol/kg, 7.5 nmol/kg, and 10 nmol/kg Sema (Novo Nordisk), after which the maximum dose was used throughout the treatment period.

For chronic TZP studies, mice were administered saline for 2 days, followed by an increasing TZP dosing paradigm (2.5 nmol/kg–10 nmol/kg) (Eli Lilly) as described with Sema.

For long-term Lira, Sema, and TZP studies, injections were administered once daily (i.p.), 1 hour prior to the onset of the dark cycle, with food intake and body weight recorded at the time of injection. All mice received a 1- to 2-day washout period with the respective vehicle after the treatment period. Body weight and food intake measurements were calculated and normalized as a fraction to the average of the pretreatment vehicle days. Cumulative food intake represents the summation of food intake during the treatment period.

### hGLP1R overexpression studies

AAV8-hsyn-DIO-hGLP1R was injected (50 nL, bilaterally) into the NTS of HFD-fed *Glp1r^Cre^* mice (NTS^GLP1R-OX^) as described above. NTS^GLP1R-OX^ or control mice were orally gavaged with vehicle (hydroxypropyl methylcellulose 2600-5600 centipoise 0.5% (w/w) and polysorbate 80 0.1% (w/w) in Millipore deionized water) twice daily for 2 days, and then orally gavaged twice daily with AZD0186 (25 mg/kg; 3 μL/g) (AstraZeneca) throughout the treatment period. The evening dose was given at the onset of the dark cycle and the morning dose 3 hours after the onset of the light cycle (6 pm/9 am). Food intake and body weight were recorded once daily in the afternoon at the time of drug administration. All mice received a 2-day washout period of twice-daily oral gavage of vehicle. Food intake and body weight were calculated and normalized as a fraction of the average measures of the pretreatment vehicle days. Cumulative food intake was calculated as the total amount of food consumed throughout the treatment period.

### Conditioned taste avoidance paradigm

For treatment of the AP^Glp1r-Dq^ and NTS^Glp1r-Dq^ mice, following an overnight fast, mice that were 8 weeks of age or older were presented with a HFD (D012492, Research Diets) and administered saline, LiCl (126 mg/kg, 203637, MilliporeSigma), or CNO (1 mg/kg). The treatments were administered 30 minutes after exposure to the HFD and HFD access was continued for another hour. On the post-conditioning day, fasted mice received access to both the HFD and chow, and the consumption of each was measured.

#### Lira.

Mice were singly housed with ad libitum access to 2 bottles containing water. For 4 days, mice received mock injections of vehicle, and the initial bottle preference was identified. Mice were then water deprived overnight at the onset of the dark cycle on day 4, and on day 5 were given 60 minutes of free access to 2 bottles containing 0.15% saccharin in water. At 60 minutes, mice were administered Lira (400 μg/kg), with continued access to saccharin for an additional 60 minutes (120 minutes total). Bottles were then removed, and water was returned to the mice at the onset of the dark cycle of the conditioning day. At the onset of the dark cycle of day 6, mice were water deprived overnight. On day 7, mice were given 2 hours’ access to 1 bottle filled with water, while the other bottle was filled with 0.15% saccharin. The preference was calculated as the volume of saccharin consumed divided by the total fluid amount consumed from both bottles. The bottle chosen to have saccharin on the post-conditioning day was the preferred bottle from the baseline drinking measurements (AP^Glp1r-Re^ studies), or the position of the water bottle and the saccharin bottle was swapped during the middle of drinking (AP^Glp1r KO^ studies).

### Gastric emptying

Mice were fasted for 4 hours prior to oral gavage of 200 μL of 20 mg/mL paracetamol in saline. Mice received an injection of 400 μg/kg Lira 1 hour prior to gavage. Fifteen minutes after gavage, we took 1 blood sample via tail vein collection. Blood was then spun at 10,000 RCF at 4°C for 10 minutes. Plasma was collected and frozen at –80°C. We used the Acetaminophen L3K detection kit (Sekure Chemistry) to detect plasma paracetamol concentrations via a Molecular Devices Spectramax m5 and reported the concentration of plasma paracetamol as mg/dL.

### Single-cell data generation

The tissue samples were processed a described in Ludwig et al. (14) using four 10X Genomics Chromium lanes and one S2 Illumina NovaSeq S2 flowcell. We followed the same protocol for generating single-nucleus suspensions, nuclei hashing, sorting, and snRNA-seq library preparation as previously described (14). Briefly, nuclei were isolated using Nuclei EZ Lysis Buffer (MilliporeSigma), subjected to an OptiPrep (MilliporeSigma) gradient for purification, and resuspended in Nuclei Buffer. Nuclei were then hashed with TotalSeq-A antibodies, stained with DAPI, and sorted using a Sony SH800S sorter with a 70 μm chip. In addition to DAPI^+^ selection, we used FACS to specifically isolate GFP^+^ nuclei for downstream analysis. Finally, snRNA-seq libraries were prepared using the Chromium Next GEM Single Cell 3′ Kit, version 3.1 (10X Genomics), following the manufacturer’s protocol, and multiplexing was performed using the BioLegend TotalSeq-A workflow.

### Single-cell RNA-seq data processing and analysis

#### Sequencing and preprocessing.

BCL files were demultiplexed into FASTQ files using bcl2fastq, version 2.19.01. RNA reads were aligned to an optimized mm10 transcriptomic reference (including intronic sequences) using Cell Ranger, version 7.0 (10X Genomics). CellBender (38) was applied to remove ambient RNA contamination. Hashtag oligo (HTO) libraries were quantified using kb count in Kallisto Bustools, version 0.24.4 (39) and processed with Seurat (40). HTOs were normalized with centered log-ratio transformation method in NormalizeData. A Gaussian mixture model was fitted to each HTO distribution, classifying droplets as positive if they belonged to the cluster with higher mean expression. Doublets were identified as droplets positive for multiple HTOs and further detected using recoverDoublets (scDblFinder). HTO^–^ cells and doublets were removed. Quality control (QC) was performed using AddPerCellQC() (Scuttle, version 1.4, Bioconductor). Cells with extreme unique molecular identifier (UMI) counts, intra- and intersample doublets, and genes expressed in fewer than 10 cells were excluded.

#### Downstream analysis.

Processing was conducted in Seurat, version 4.3.0. For each 10X Genomics lane, cells with fewer than 1,000 UMI counts and fewer than 1,000 unique genes were identified and removed. Raw counts were normalized using NormalizeData, and principal component analysis (PCA) was performed with RunPCA, using the top 3,000 variable genes as input. Clustering was then conducted using FindNeighbors and FindClusters, with the top 30 principal components (PCs) as input.

Among 47,526 quality controlled cells, we first removed glial cells and subsequently renormalized the data, performed PCA, and reclustered the dataset. Following glia removal, the dataset contained 43,919 cells across 15 samples.

We conducted label transfer using an internal dataset to distinguish dorsal vagal complex (DVC) cells from hypothalamic (Hypo) cells (41). A threshold for prediction confidence was deemed unnecessary, as approximately 27,500 of the 43,919 cells had a prediction score above 0.9, and the remaining cells aligned with expected locations when no threshold was applied.

To further refine the analysis, we subsetted the DVC fraction and conducted label transfer with a DVC atlas, applying a prediction score threshold of 0.8. AP cells were then identified on the basis of our spatial transcriptomics dataset (14), in which the following clusters were classified as AP cells: “GABA_0_0,” “GABA_0_2,” “Glu_2_1,” “Glu_5_1,” “Glu_5_2,” and “Glu_6_1,” with Glu_2_1, Glu_5_1, and Glu_5_2.

For differential expression analysis, we pseudobulked the dataset by aggregating AP and non-AP cells for each sample. The following linear regression model was applied: model.matrix (~ sample + cluster, data = sun1_pseudo$samples), where the cluster term differentiates AP versus non-AP cells. Genes were considered significantly differentially expressed on the basis of an FDR threshold below 0.05. Genes were then ranked by the percentage at which they were the top-10 average log_2_ fold change). The percentage of expressing cells for each gene within the 2 groups (AP vs. non-AP) was extracted from Seurat’s FindMarkers function and visualized.

To determine whether the observed differential expression was specific to the DVC or also present in the hypothalamus, we mapped the DVC labels back onto the original dataset and revisualized the expression patterns.

### Perfusion and IHC analysis

At the conclusion of all experiments, mice were deeply anesthetized with isoflurane and transcardially perfused with PBS followed by 10% buffered formalin. Brains were removed, placed in 10% buffered formalin for 4–24 hours, and dehydrated in 30% sucrose for at least 48 hours. Using a freezing microtome (Leica), brains were cut into 30 μm sections and collected in 4 series.

For all IHC procedures, sections were blocked for 60 minutes (PBS with 0.03% Triton X-100, 3% normal donkey serum). The sections were incubated overnight (12–24 hours) at room temperature (RT) using primary antibodies (rabbit anti–GLP-1R, EPR21819 [mGLP1R] or EPR23507 [hGLP1R], Abcam, 1:500; rabbit anti-FOS, 2250, Cell Signaling Technology, 1:1,000; mouse anti-CGRP, ab81887, Abcam, 1:1,000; chicken anti-GFP, GFP1020, Aves Laboratories, 1:1,000). Tissue-bound antibodies were detected with species-specific Alexa Fluor 488–, Alexa Fluor 568–, or Alexa Fluor 647–conjugated secondary antibodies (Invitrogen, Thermo Fisher Scientific, 1:250). Images were collected on an Olympus BX53F microscope.

For FOS counts, detectable FOS^+^ soma were counted in the AP of animals treated with saline (AP^Ctrl^) or Lira (AP^GLP1R-KO^) (400 μg/kg, i.p.). The numbers of FOS^+^ soma per section in the AP were averaged and compared across groups.

### ISH

Brains embedded with Fisher Healthcare Tissue-Plus O.C.T. Compound (Thermo Fisher Scientific, catalog 23-730-571) were transferred from a –80°C freezer to the Leica Biosystems CM 1860 Cryostat and equilibrated to –20°C for 1 hour before being sliced into 16 μm sections onto Epredia Superfrost Plus Adhesion Control Microscope Slides (Thermo Fisher Scientific, catalog 22-042-941) in an RNAse-free environment. Slides were stored at –80°C and used within 3 months of sectioning.

Slides were stained using our adaptation of the RNAscope 2.5 HD RED assay protocol by Advanced Cell Diagnostics (ACD) using reagents supplied by ACD, unless otherwise indicated. Slides were placed in Sigma-Aldrich 10% neutral-buffered formalin (NBF) (catalog HT501128) solution for approximately 75 minutes at 4°C and were then placed in ethanol solutions (50%, 70%, 100%, and 100% again). After drying for 5 minutes, a hydrophobic barrier was created around the sections on the slides using a Vector Laboratories ImmEdge Hydrophobic Barrier PAP Pen (catalog H-4000). This barrier was allowed to dry for approximately 1 minute, after which 4 drops of RNAscope Hydrogen Peroxide (ACDbio, catalog 322335) were applied for 10 minutes to the sections on each slide. Slides were washed in PBS Buffer for approximately 2 minutes before applying 4 drops of RNAscope Protease III (ACDbio, catalog 322337) to each slide. Next, the slides were washed twice with fresh PBS before pipetting 130 μL RNAscope Probe – Mm-Glp1r-O2-C1 (catalog 1572621-C1) onto each slide. The slides were then incubated for 2 hours at 40°C using the ACD HybEZ II Hybridization System (catalog 321711).

Following hybridization, slides were washed twice with a fresh RNAscope 1× Wash Buffer and placed in a 5× saline sodium citrate (SSC) buffer (diluted from Thermo Fisher Scientific, 20× SSC buffer, catalog J60839.K2) overnight. The next day, the slides were washed again twice with RNAscope Wash Buffer. For each amplification reagent (RNAscope 2.5 HD RED AMP 1-6, from RNAscope 2.5 HD Detection Reagents-RED assay, catalog 322360), 130 μL was pipetted onto each slide and incubated in the humidity control tray from the ACD HybEZ II Hybridization System. After each incubation period, the slides are washed twice with fresh RNAscope Wash Buffer. During their incubation periods, AMP 1-4 were also placed inside the ACD HybEZ II oven at 40°C. AMP 5-6 were incubated at RT. The incubation length for AMP 1, 3, and 5 was 30 minutes, and for AMP 2, 4, 6, it was 15 minutes. Next, 120 μL of a 120:2 mixture of RNAscope Fast Red A and RNAscope Fast Red B (both from RNAscope 2.5 HD Detection Reagents-RED assay, catalog 322360) were pipetted onto each slide. The slides were incubated for 10 minutes in the humidity control tray at RT for 10 minutes and then washed twice with fresh tap water. Slides were coverslipped with Invitrogen Fluoromount-G Mounting Medium (Thermo Fisher Scientific, catalog 00-4958-02) and Fisherbrand 60 × 22 mm Microscope Cover Glass (catalog 12-545-JP). After letting the mounting medium solidify for approximately 1 day, the slides were visualized using the fluorescence microscope, Olympus BX53 (catalog BX53-DIC-FL).

### Statistics

All plotting and statistical analyses were performed using GraphPad Prism (GraphPad Software). Specific statistical tests (e.g., 1- or 2-way ANOVA) are listed in the figure legends, along with sample sizes. One-way ANOVA was used for comparisons among single measurements for each of three or more groups; 2-way ANOVA was used for multiple comparisons (e.g., measurements at multiple time points) for each of three or more groups. All *t* tests were 2 tailed. A *P* value of less than 0.05 was considered significant.

### Study approval

All animal experiments were performed according to the protocols approved by the appropriate IACUCs of Zhejiang University (approval no. 23311) and the University of Michigan (approval no. 11066) and, at the University of Michigan, were conducted in accordance with Association for the Assessment and Approval of Laboratory Animal Care and National Institutes of Health guidelines.

### Data availability

Tables with all primary rodent data values (primary data) are included in the Supporting Data Values file. RNA-seq data are available at the European Bioinformatics Institute (EBI), ArrayExpress (accession E-MTAB-17566; https://www.ebi.ac.uk/biostudies/arrayexpress/studies/E-MTAB-17566?key=50306a89-83c6-49cb-a64e-7272197fb3bc). All code can be found at https://github.com/perslab/warren-2025; commit ID 3a5f389. All data are included in the manuscript and supplemental files and/or have been deposited online.

## Manuscript information

### Author contributions.

WTY, WQ, and MGM conceived the project, designed experiments, and analyzed data. WTY, YW, GZ, SH, CB, EW, JGS, FS, MD, SK, IW, JMB, DBR, ACR, AJT, ZL, EV, THP, and WQ performed experiments and analyzed data. WQ and GZ performed all tetanus toxin, hM3Dq DREADD, and *Glp1r^fl^* studies. ZL analyzed data and provided animal care. AS, KR, and RJS aided in project and experimental design and provided reagents. WTY and MGM wrote the manuscript with input from all authors. MGM and WQ acquired funding.

### AI disclosure.

No AI was used in the preparation of this manuscript.

### Conflict of interest.

MGM receives research support from AstraZeneca, Eli Lilly, and Novo Nordisk. MGM has served as a paid consultant for Merck, Third Rock, and Zealand Pharma and has received speaking honoraria from Novo Nordisk. RJS has received research support from Novo Nordisk, Fractyl, Astra Zeneca, Congruence Therapeutics, Eli Lilly, Bullfrog AI, Glycsend Therapeutics, and Amgen. RJS has served as a paid consultant for Novo Nordisk, Eli Lilly, CinRx, Fractyl, Structure Therapeutics, Crinetics, and Congruence Therapeutics. RJS has equity in Calibrate, Rewind Therapeutics, and Levator Therapeutics. AS and KR work for and hold equity in Novo Nordisk. THP has received consulting fees from Eli Lilly and Zealand Pharma, receives research support from Novo Nordisk, and his household owns Novo Nordisk stock. JS owns Novo Nordisk stock.

### Funding.

This study was supported by the following:

- The Novo Nordisk Foundation Center (NNFC) for Basic Metabolic Research is an independent research center based at the University of Copenhagen and is partially funded by an unconditional donation from the Novo Nordisk Foundation (NNF) (https://www.cbmr.ku.dk/).
- NIH (grant R01DK141495, to MGM).
- NIH (grant F31DK142280, to WTY).
- NIH (grant DK124238, to THP).
- Novo Nordisk (grant to THP and MGM).
- NNF (grant NNF18OC0033444, to the Center for Adipocyte Signaling and to FS).
- NNFC (grant 32371212, to WQ).
- NNFC (grant NNF18CC0034900).
- Novo Nordisk, NNF (grant NNF16OC0021496, to THP).
- Lundbeck Foundation (grant R190-2014-3904, to THP).

This work is the result of NIH funding, in whole or in part, and is subject to the NIH Public Access Policy. Through acceptance of this federal funding, the NIH has been given a right to make the work publicly available in PubMed Central.

## Supplementary Material

Supplemental data

Supporting data values

## Figures and Tables

**Figure 1:**
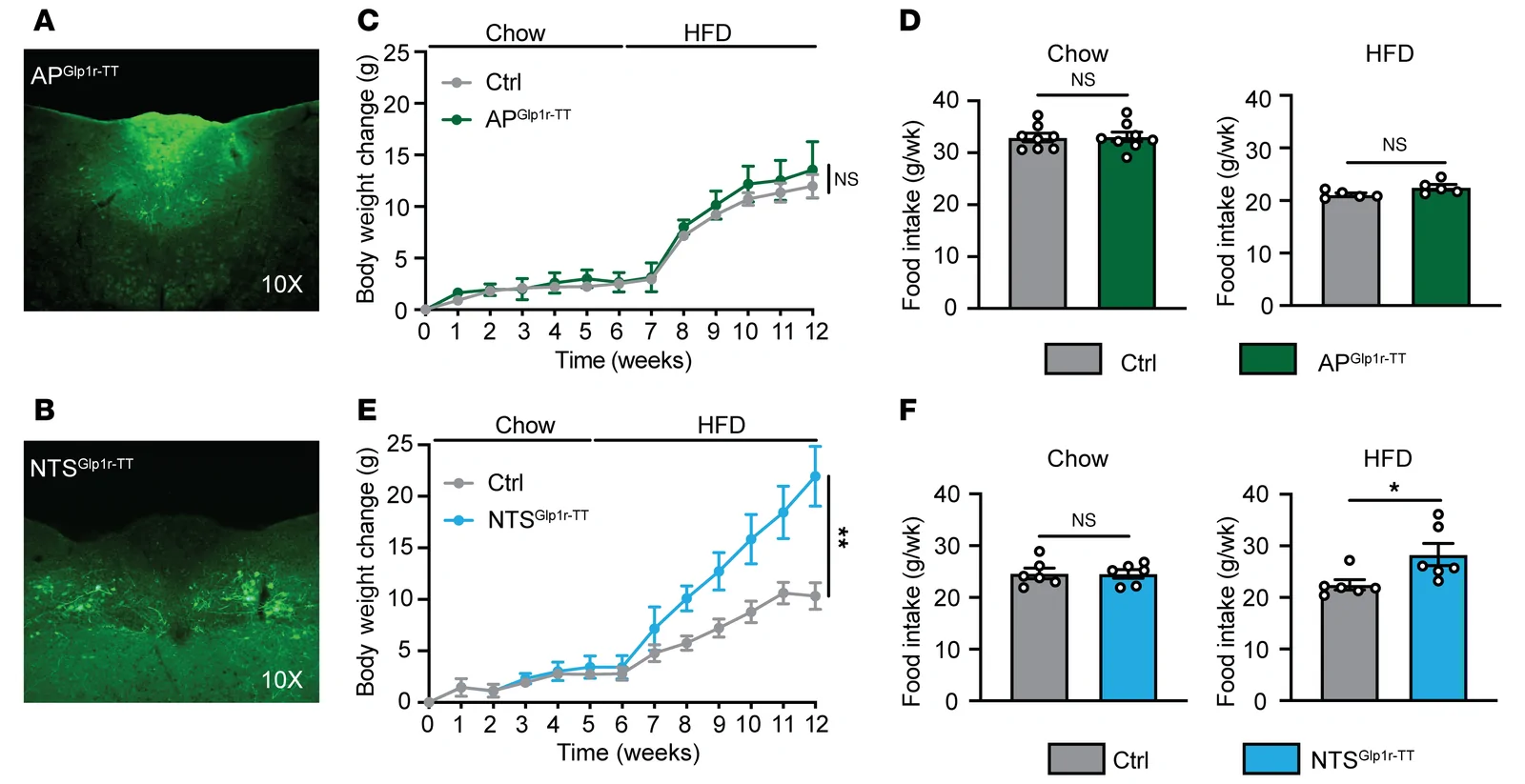
Silencing NTS^Glp1r^ neurons increases food intake and body weight during HFD feeding. (**A** and **B**) Representative images of GFP IR (green) in the DVC of AP^Glp1r-TT^ (**A**) and NTS^Glp1r-TT^ (**B**) mice. Original magnification, ×10 (cropped). (**C**) Body weight change following surgery for AP^Glp1r-TT^ (*n* = 4; 2 males, 2 females) and control (Ctrl) (*n* = 10; 4 males, 6 females) mice on a chow diet for 6 weeks followed by a HFD for 6 weeks. (**D**) Weekly food intake for AP^Glp1r-TT^ (*n* = 5–8; 2–4 males, 3–4 females) and control (*n* = 5–8; 2–4 males, 3–4 females) mice during the final week of chow or HFD feeding. (**E**) Body weight change compared with week 1 following surgery for NTS^Glp1r-TT^ (*n* = 4 males) or control (*n* = 5 males) mice on chow for 5 weeks followed by a HFD for 7 weeks. (**F**) Food intake of NTS^Glp1r-TT^ (*n* = 6 males) or control (*n* = 6 males) mice during the final week of chow or HFD feeding. All data are presented as the mean ± SEM. Repeated-measures 2-way ANOVA with Šídák’s multiple comparisons for **C** and **E**. Significance in **E** is the ANOVA interaction effect. Unpaired, 2-tailed *t* test with Welch’s correction for **D** and **F**. NS = *P* > 0.05; *P < 0.05.

**Figure 2:**
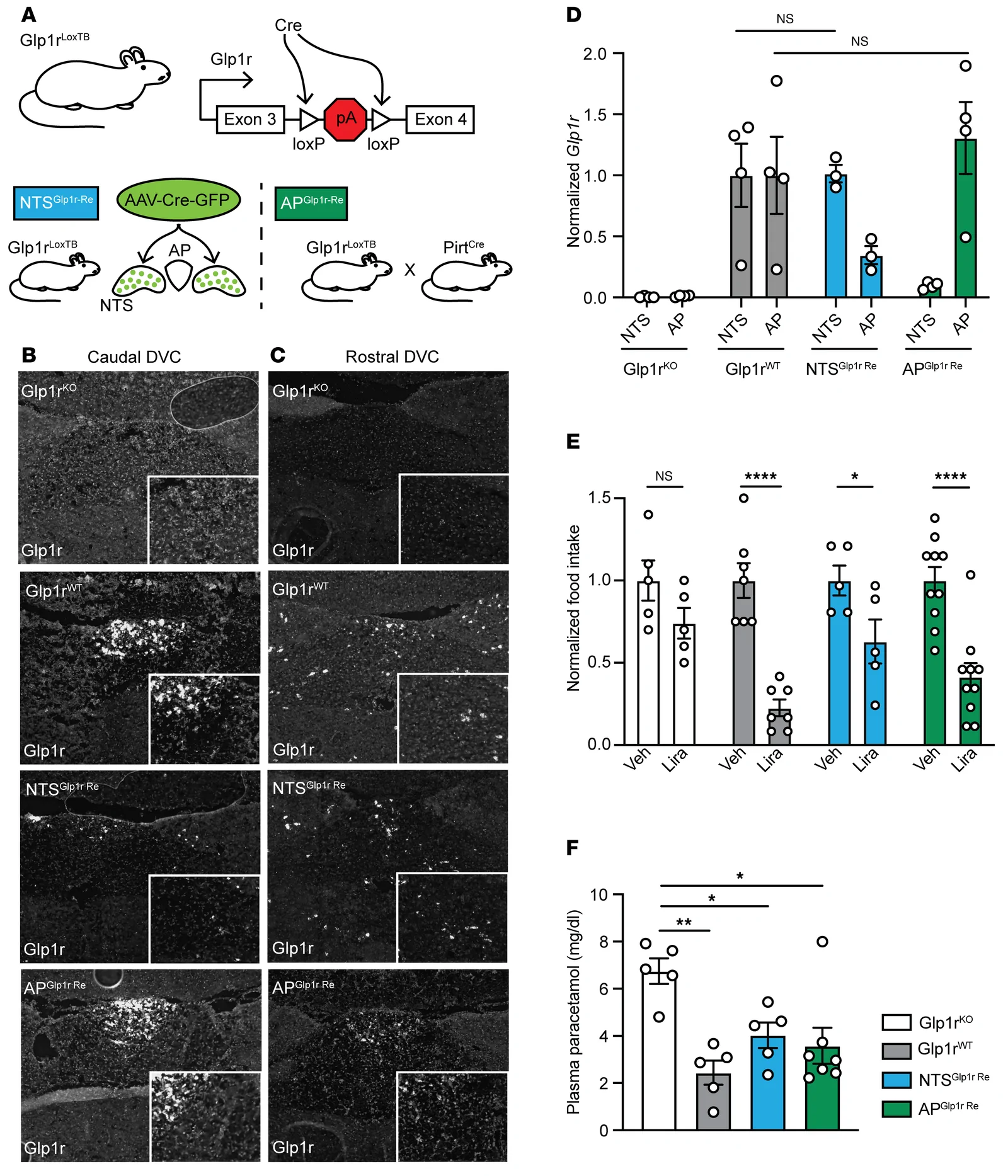
AP^Glp1r-Re^ and NTS^Glp1r-Re^ mice and their acute responses to Lira. (**A**) Schematic showing the genetics of NTS^Glp1r^ ^Re^ and AP^Glp1r^ ^Re^ mice. (**B** and **C**) Representative images of ISH for *Glp1r* in Glp1r^WT^, Glp1r^KO^, NTS^Glp1r^ ^Re^, and AP^Glp1r^ ^Re^ mice in the rostral (**B**) and caudal (**C**) DVC. Original magnification ×10; insets are digital zooms of the original images. (**D**) Quantification of *Glp1r* detection in the DVC across all mouse models (Glp1r^KO^, *n* = 4; 2 males, 2 females; Glp1r^WT^, *n* = 4; 2 males, 2 females; NTS^Glp1r^ ^Re^, *n* = 3; 2 males, 1 females; AP^Glp1r^ ^Re^, *n* = 4; 3 males, 1 female). Data for each anatomic region were normalized to *Glp1r* expression from that region in Glp1r^WT^ animals. (**E**) HFD intake 4 hours after saline (vehicle [Veh]) or Lira (400 μg/kg, i.p.) injection. Food intake values were normalized to average 4-hour food intake in vehicle-treated mice. Glp1r^KO^ (*n* = 5 females), Glp1r^WT^, (*n* = 7; 3 males, 4 females), NTS^Glp1r-Re^ (*n* = 5; 2 males, 3 females), and AP^Glp1r-Re^ (*n* = 10; 5 males, 5 females) animals. (**F**) Plasma paracetamol concentrations 15 minutes after gavage of paracetamol in saline for Lira-treated Glp1r^KO^ (*n* = 5 females), Glp1r^WT^ (*n* = 5; 3 males, 2 females), NTS^Glp1r-Re^ (*n* = 5; 2 males, 3 females), and AP^Glp1r-Re^ (*n* = 7; 5 males, 2 females) mice. All graphs show the mean ± SEM. All DVC images are ×10 pseudocolored black and white. Two-way ANOVA with Tukey’s multiple comparisons for **D** and **E**. One-way ANOVA with Tukey’s multiple comparisons for **F**. NS = *P* > 0.05; *P < 0.05, **P < 0.01, and ****P < 0.0001.

**Figure 3:**
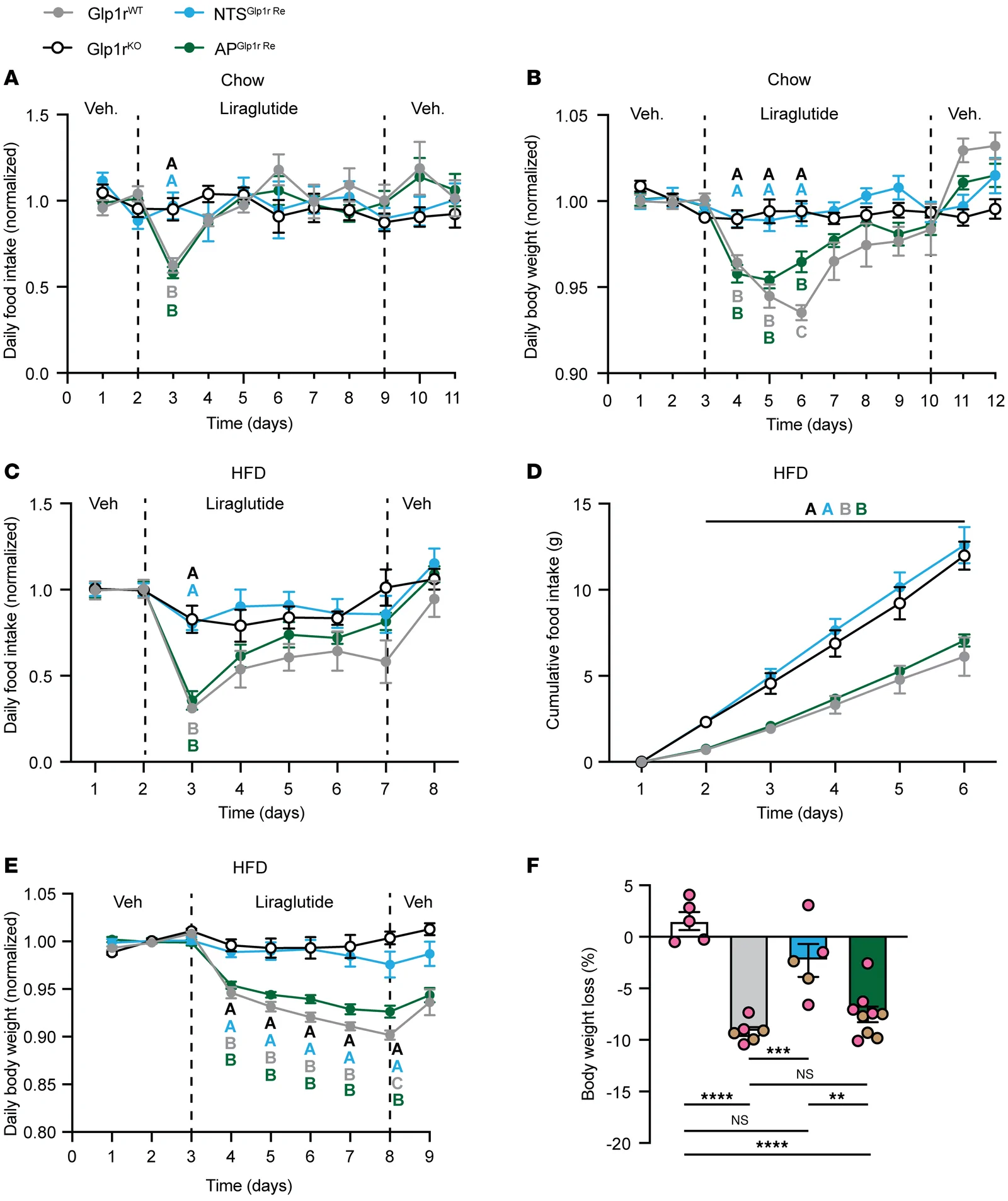
Lira reduces food intake and body weight of AP^Glp1r-Re^ but not NTS^Glp1r-Re^ mice. The indicated strains of mice were injected with vehicle for 2 days, Lira for 5–7 days, and vehicle again for 1–2 days; food intake and body weight were measured daily. (**A** and **B**) Food intake (**A**) and body weight (**B**) were normalized to baseline for chow-fed Glp1r^KO^ (*n* = 5–6), Glp1r^WT^ (*n* = 6–7), NTS^Glp1r-Re^ (*n* = 5), and AP^Glp1r-Re^ (*n* = 9–10) mice. (**C** and **D**) Daily food intake for HFD-fed mice. (**C**) Daily food intake was normalized to baseline for vehicle- and Lira-treated mice and (**D**) cumulative food intake was measured over the course of Lira treatment. Daily body weight was normalized to baseline (**E**) and to the total percentage of body weight change (**F**) during Lira treatment for HFD-fed Glp1r^KO^ (*n* = 5), Glp1r^WT^ (*n* = 6), NTS^Glp1r-Re^ (*n* = 5), and AP^Glp1r-Re^ (*n* = 9) mice. Pink circles in **F** indicate data for female mice; brown circles indicate data for male mice. All graphs show the mean ± SEM. Repeated-measures, 2-way ANOVA with Tukey’s multiple comparisons for **A**–**E**. One-way ANOVA with Tukey’s multiple comparisons for **F**. **A**–**E**: Groups (indicated by the letter matching the color of the line) with different letters are statistically significant at the indicated time point, *P* < 0.05. **F**: NS = *P* > 0.05; **P < 0.01, ***P < 0.001, and ****P < 0.0001.

**Figure 4:**
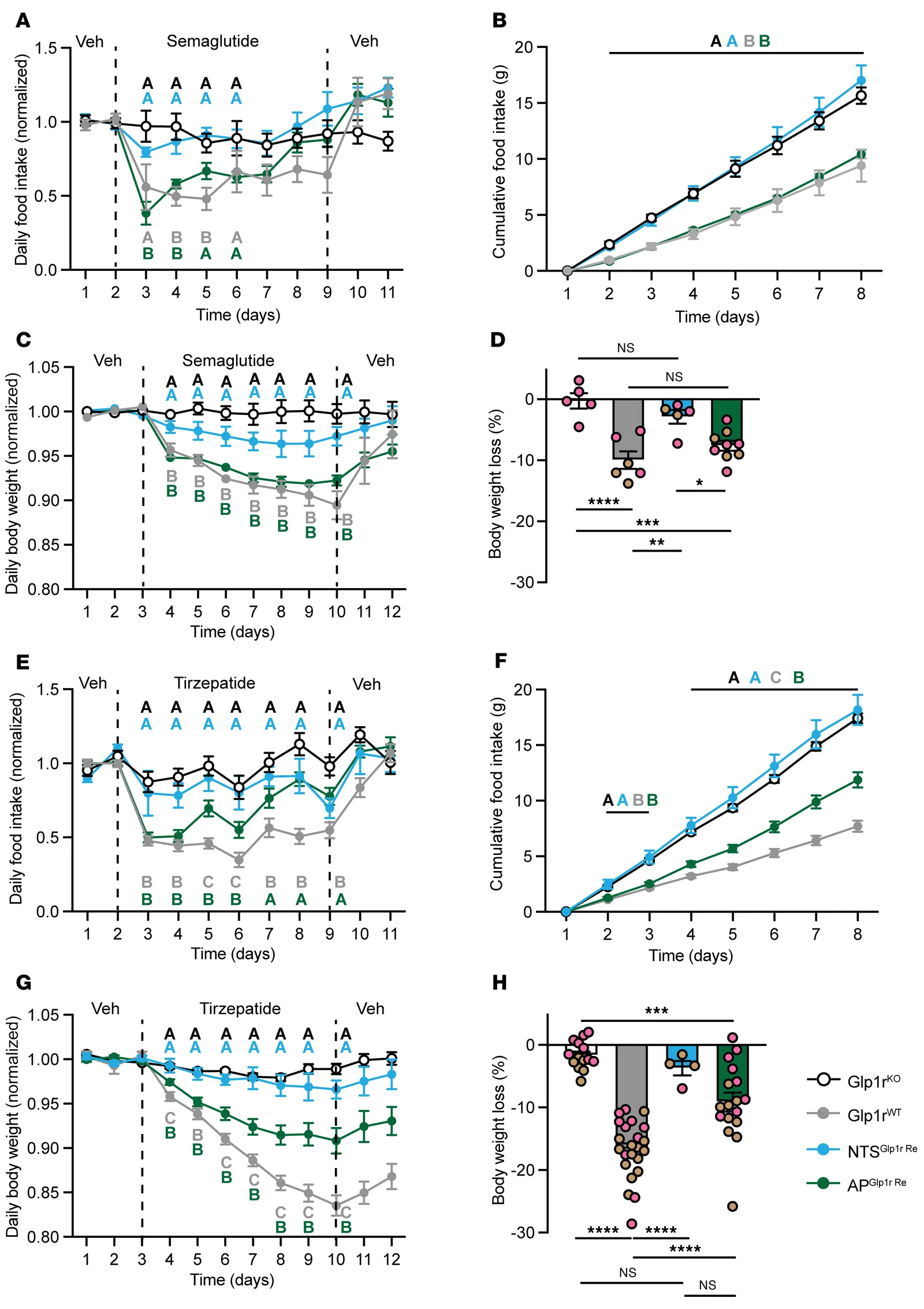
Effects of Sema and TZP on food intake and body weight in AP^Glp1r-Re^ and NTS^Glp1r-Re^ animals. Animals were treated with vehicle for 2 days, Sema for 7 days, and vehicle for 2 additional days. Food intake and body weight were measured once daily. Shown are daily food intake over the course of the experiment (**A**), cumulative food intake during Sema treatment (**B**), daily body weight (**C**), and overall change in body weight (**D**) during Sema treatment for HFD-fed Glp1r^KO^ (*n* = 5), Glp1r^WT^ (*n* = 6), NTS^Glp1r-Re^ (*n* = 5), and AP^Glp1r-Re^ (*n* = 9) animals. For TZP treatment, mice were treated with vehicle for 2 days, TZP for 7 days, and vehicle for 2 additional days. Food intake and body weight were measured once daily. Shown are daily food intake over the course of the experiment (**E**), cumulative food intake during TZP treatment (**F**), daily body weight (**G**), and overall change in body weight (**H**) during TZP treatment for HFD-fed Glp1r^KO^ (*n* = 13–14), Glp1r^WT^ (*n* = 23), NTS^Glp1r-Re^ (*n* = 4), and AP^Glp1r-Re^ (*n* = 16–17) animals. Pink circles indicate data from female mice; brown circles indicate data from male mice. Data in **A**, **C**, **E**, and **G** were normalized to baseline measurements. All graphs show the mean ± SEM. Repeated-measures, 2-way ANOVA with Tukey’s multiple comparisons for **A**–**C** and **E**–**G**. One-way ANOVA with Tukey’s multiple comparisons for **D** and **H**. **A**–**C**, and **E**–**G**: Groups (indicated by the letter matching the color of the line) with different letters differ at the indicated time point, *P* < 0.05. **D**: NS = *P* < 0.05; **F**: NS = *P* > 0.05; *P < 0.05, **P < 0.01, ***P < 0.001, and ****P < 0.0001.

**Figure 5:**
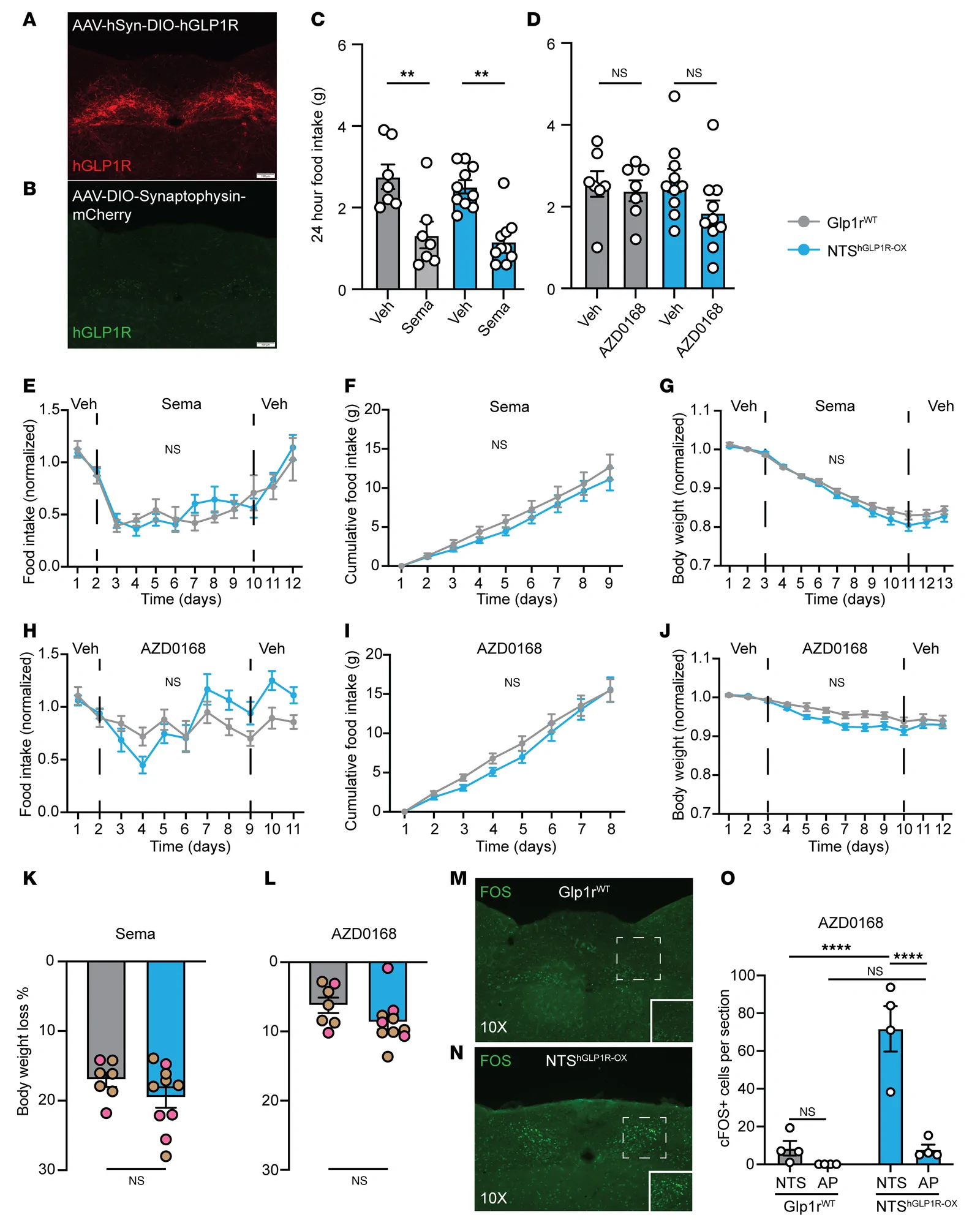
Overexpression of *hGLP1R* in NTS^Glp1r^ neurons does not alter the long-term response to GLP1Ras. (**A**) Example DVC image of hGLP1R IR in NTS^OX-hGLP1R^ mice. (**B**) Example image of hGLP1R IR in Glp1r^WT^ mice. Scale bars: 100 μm. (**C** and **D**) Food intake over a 24-hour period in Glp1r^WT^ (*n* = 7) and NTS^OX-hGLP1R^ mice (*n* = 10) treated with Sema (**C**) or AZD0186 (**D**). (**E**–**J**) Glp1r^WT^ (*n* = 7) and NTS^OX-hGLP1R^ mice (*n* = 10) were treated once daily with Sema (**E**–**G**) or twice daily with AZD0186 (**H**–**J**). Shown for the Sema study are daily food intake values normalized to vehicle baseline days (**E**), cumulative food intake throughout the treatment period (**F**), and daily body weight normalized to vehicle baseline values (**G**). Shown for the study with AZD0186 are daily food intake values normalized to vehicle baseline days (**H**), cumulative food intake throughout the treatment period (**I**), and daily body weight normalized to vehicle baseline values (**J**). Total body weight loss following Sema (**K**) or AZD0186 (**L**) treatment in Glp1r^WT^ (*n* = 7) and NTS^OX-hGlp1r^ mice (*n* = 10). Pink circles indicate data for female mice; brown circles indicate data for male mice. (**M** and **N**) Example images of FOS IR (green) in the DVC for AZD0186 in Glp1r^WT^ (**M**) or NTS^OX-hGLP1R^ (**N**) mice, with digital zoom insets of the NTS (Original magnification, ×10). (**O**) Quantification of FOS IR in the NTS from the samples (*n* = 4 each) represented in **M** and **N**. Two-way ANOVA with Tukey’s multiple comparisons for **C**, **D**, and **O**; repeated-measures, 2-way ANOVA with Tukey’s multiple comparisons for **E**–**J**; unpaired, 2-tailed *t* test with Welch’s correction for **K** and **L**. NS = *P* < 0.05; **F**: NS = *P* > 0.05; **P < 0.01 and ****P < 0.0001.

## Version 1 09/15/2026

In-Press Preview

## Version 2 11/02/2026

Electronic publication
