# Supplementary material for: A single dorsal vagal complex circuit mediates the aversive and anorectic responses to GLP1R agonists
Source: J Clin Invest. 2026 Sep 15;136(21):e193743. doi: 10.1172/JCI193743 (PMC13722397; doi:10.1172/JCI193743)
Supplement: Supplementary file 1 [file jci-136-193743-s115.pdf]

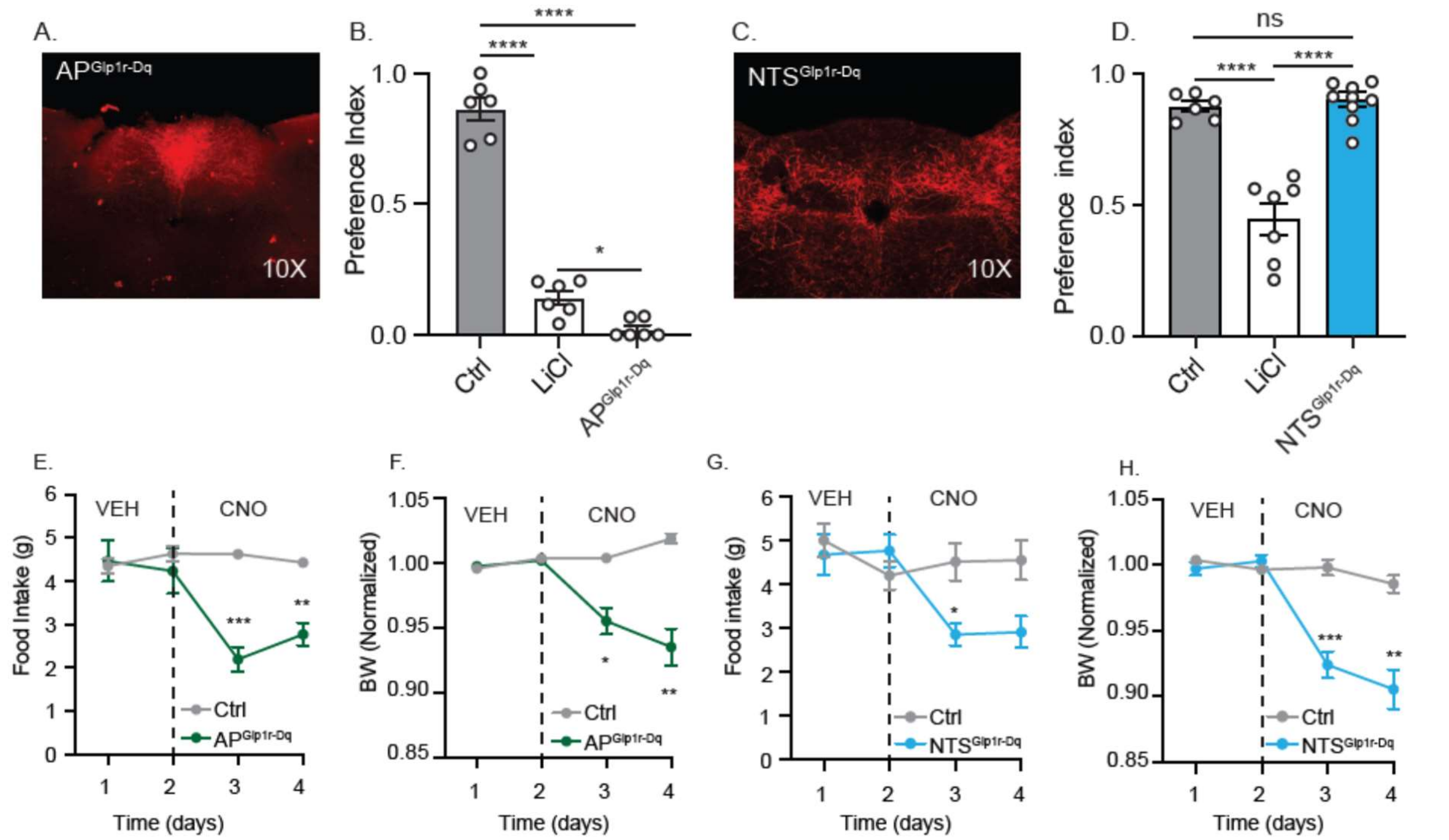

Supplemental Figure 1: Activating AP^{Glp1r} or NTS^{Glp1r} neurons suppresses food intake, but only AP^{Glp1r} cells promote CTA formation. (A, C) Example images of mCherry-IR (red) in the DVC of $AP^{Glp1r-Dq}$ (A) and $NTS^{Glp1r-Dq}$ (C) mice (images acquired at 10x magnification). (B) Preference index for HFD in $AP^{Glp1r-Dq}$ mice treated with saline (Ctrl, n=6), LiCl (126 mg/kg) (n=5), or CNO (1mg/kg, n=6). (D) Preference index for HFD in $NTS^{Glp1r-Dq}$ mice treated with saline (CTRL, n=6), LiCl (126 mg/kg) (n=7), or CNO (1mg/kg, n=9). (E-F) Control (n=6) and $AP^{Glp1r-Dq}$ (n=6) mice were given a loading dose of CNO (1mg/kg, IP) followed by 48-hour *ad libitum* access to CNO in a water bottle while monitoring food intake (E) and body weight (F) daily. (G-H) Control (n=6) and $NTS^{Glp1r-Dq}$ (n=9) mice were given a loading dose of CNO (1mg/kg, IP) followed by 48-hour *ad libitum* access to CNO in a water bottle while monitoring food intake (G) and body weight (H) daily. Body weight in F, H are normalized to baseline. All graphs: mean +/- SEM is shown. One way ANOVA with Tukey's test for B-C. Repeated measures two-way ANOVA with Sidaks multiple comparisons for E-H. NS $p>0.05$, *$p<0.05$, **$p<0.01$, ***$p<0.001$, ****$p<0.0001$.

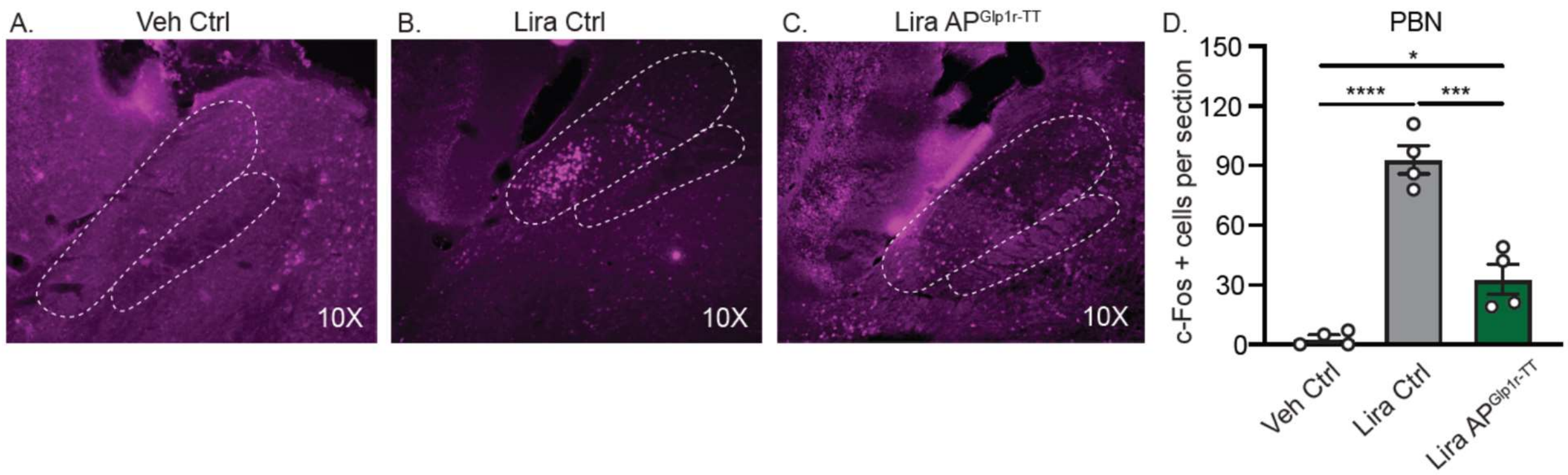

Supplemental Figure 2: Silencing AP^{Glp1r} neurons blocks Lira-induced PBN FOS
(A) Representative images of FOS-IR in the PBN of Ctrl mice treated with saline (Veh), (B) Lira (400 ug/kg, IP), or (C) $AP^{Glp1r\text{-}TT}$ mice treated with Lira (10X mag.). (D) Quantification of PBN FOS-IR cells for mice treated as in (A-C, n=4 each). Mean +/- SEM is shown. One way ANOVA with Tukey's test. ns- p>0.05, *p<0.05, **p<0.01, ***p<0.001, ****p<0.0001.

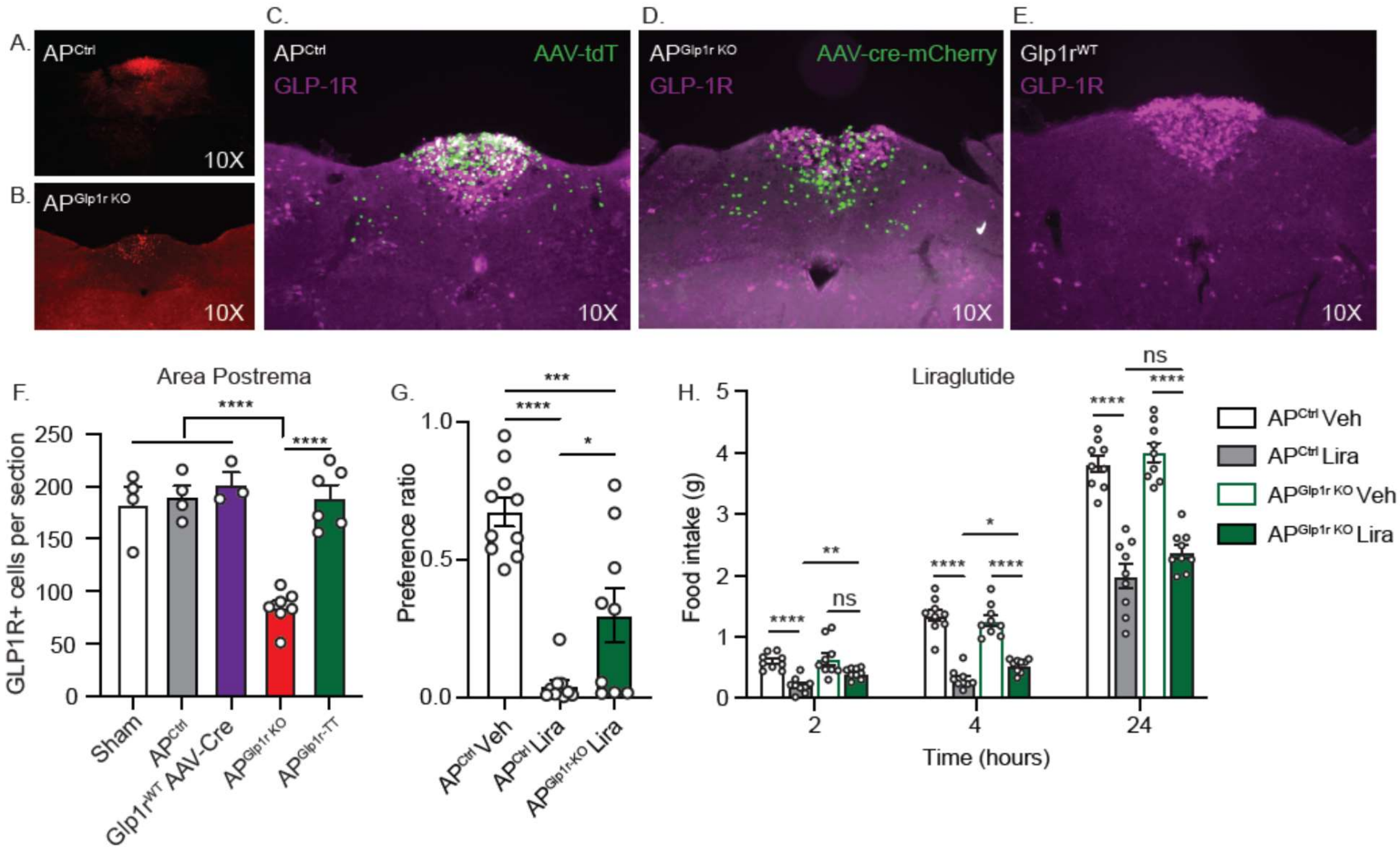

Supplemental Figure 3: AP^{Glp1r} neurons contribute to the anorectic and aversive responses to Lira

(A-B) Representative images showing mCherry-IR (red) in the DVC of AP^{Ctrl} (A) and AP$^{Glp1r\text{-}KO}$ (B) mice (images acquired at 10X magnification). (C-E) Representative 10X images of GLP1R-IR (magenta) and mCherry or tdTomato-IR (pseudocolored green)in the AP of (C) AP^{Ctrl}, (D) AP$^{Glp1r\ KO}$, and (E) Glp1r^{WT} mice injected with AAV-Cre-mCherry (mCherry-IR not shown for E). (F) Quantification of AP GLP1R-IR for animals of the indicated type (n=3-7 per group). (G) Preference index calculated for saccharin in AP$^{Glp1r\ KO}$ (n=9) or AP^{Ctrl} (n=10 each treatment) animals treated with vehicle (Veh) or Lira (400 ug/kg, IP), as indicated. (H) Cumulative food intake 2, 4, and 24 hours after the onset of the dark cycle for AP^{Ctrl} (n=9) and AP$^{Glp1r\text{-}KO}$ (n=9) animals following vehicle (Veh) or Lira (400ug/kg, IP) injection. One-way ANOVA w/Tukey's multiple comparisons for (F, G). Repeated measures two-way ANOVA w/Tukey's multiple comparisons for H. All graphs: mean +/- SEM is shown. ns- $p>0.05$, *$p<0.05$, **$p<0.01$, ***$p<0.001$, ****$p<0.0001$.

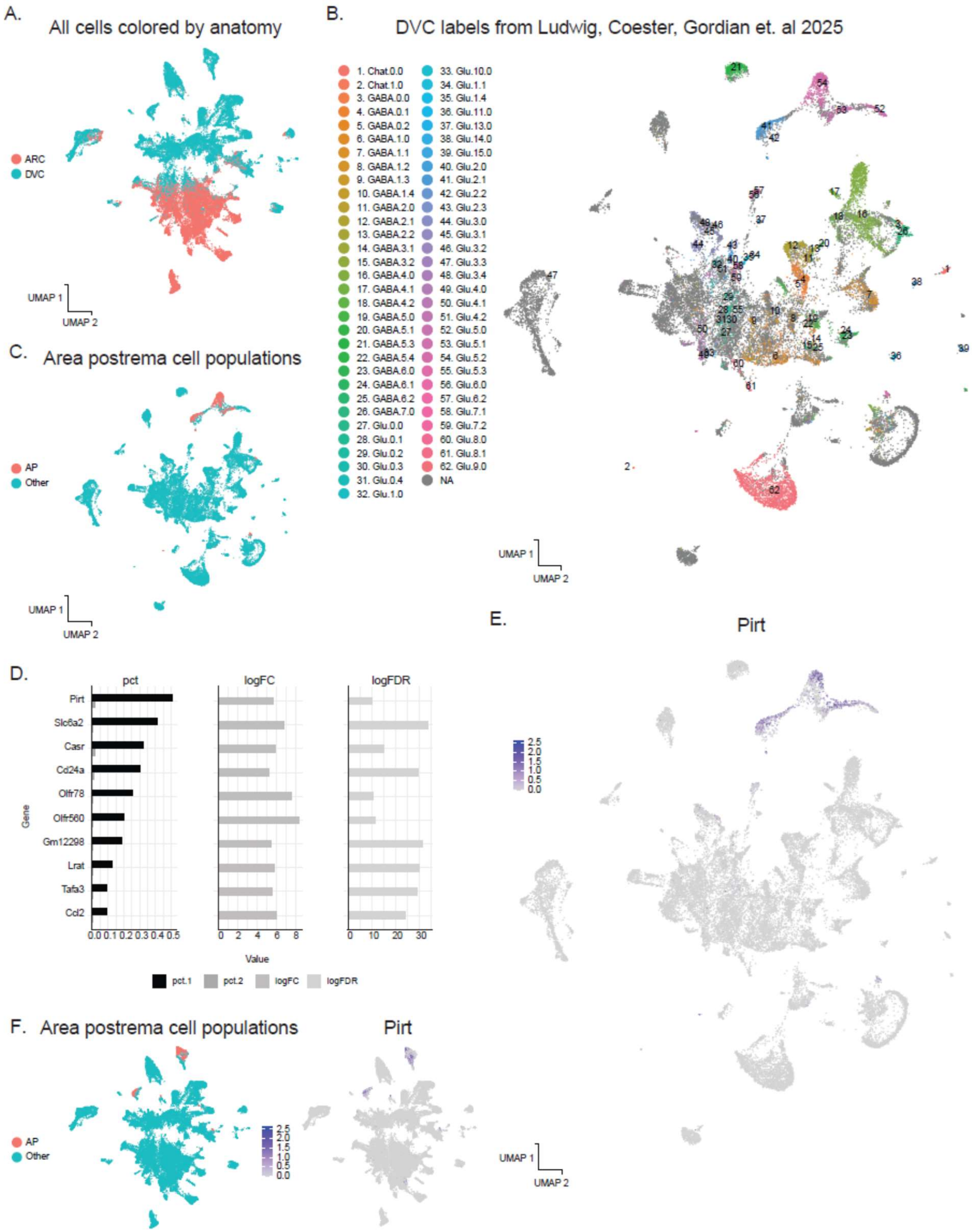

Supplemental Figure 4: Identification of *Pirt* as a marker gene for AP[Glp1r] neurons.
(A) Label transfer of ARC and DVC dataset as used to separate 43919 SUN1-GFP-contianing cells (n=15) into DVC and mediobasal hypothalamic (ARC) cell populations (comprising 26131 and 17788 cells, respectively). (B-C) DVC atlas (14) label transfer

reveal distinct AP-specific clusters in the DVC fraction of SUN1-GFP-containing cells. (D) DEG analysis of AP cells highlight *Pirt* as a potent AP marker gene. Top 10 DE genes (sorted by logFC) are shown. ”Pct.1” and ”Pct.2” denotes the percentage of cells where the gene is detected in the area postrema cell populations and other cell populations, respectively. Abbreviations: FDR, false-discovery rates; log, logarithm base 10**.** (E) *Pirt* is selectively expressed in AP cell clusters. (F) *Pirt* expression is limited in the hypothalamus.

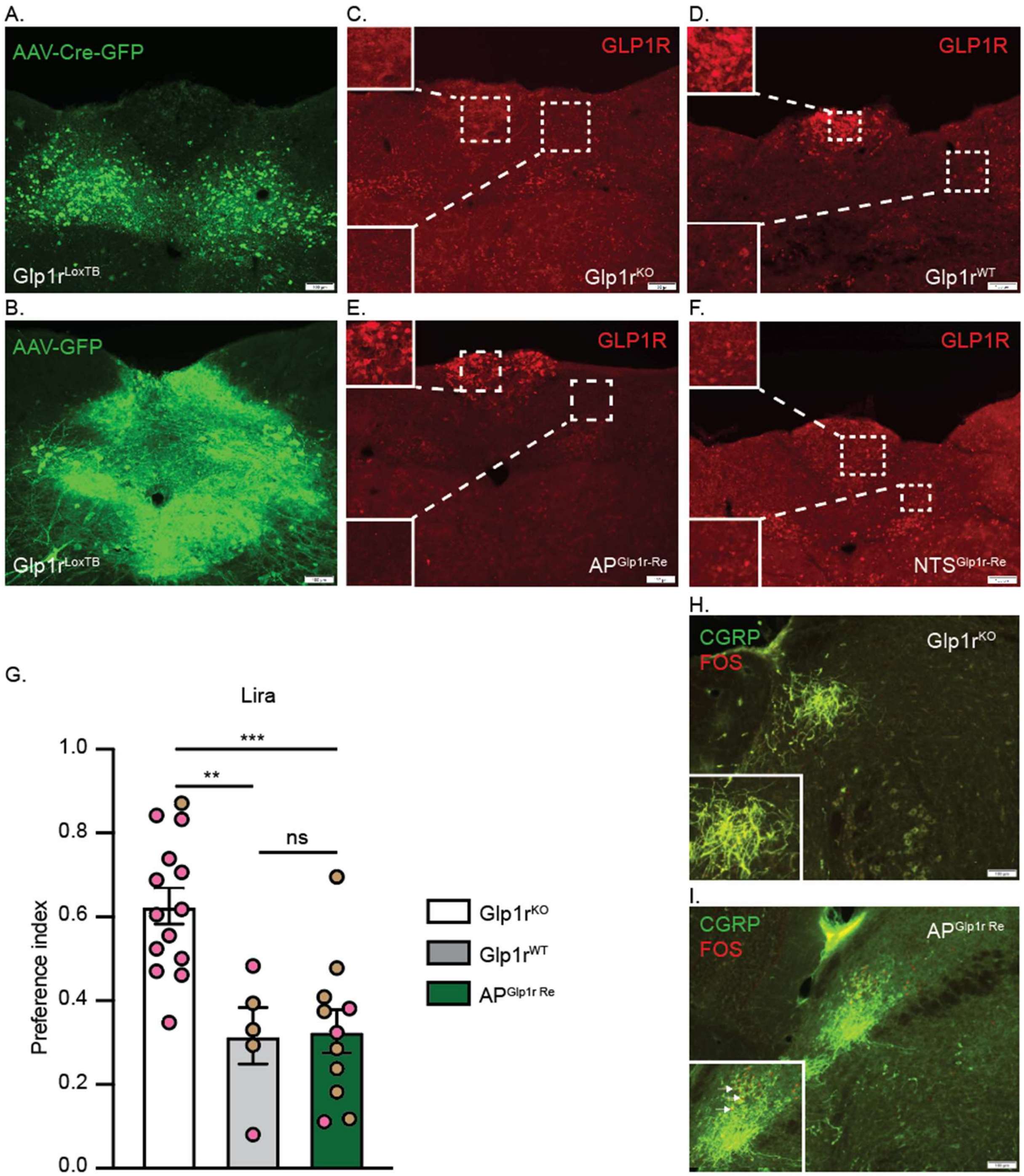

<u>Supplemental Figure 5: Validating AP$^{Glp1r\ Re}$ and NTS$^{Glp1r\ Re}$ mice</u>

(A-B) Example images of GFP-IR in the DVC of *Glp1r$^{LSL/LSL}$* mice following intra-NTS injection of AAV-Cre-GFP or AAV-GFP, scale bar= 100um.C-F: Representative images of GLP1R-IR (red) in the DVC of control Glp1r^{KO} (A), Glp1r^{WT}, (B), AP$^{Glp1r\text{-}Re}$ (C), and NTS$^{Glp1r\text{-}Re}$ (D) mice (n=3 each scale bar=100 um). Inserts represent digital zoom of indicated areas. (G) Preference index for saccharin in Glp1r^{KO} (n=14), Glp1r^{WT} (Ctrl; n=5), or AP$^{Glp1r\text{-}Re}$ (n=11) mice treated with liraglutide (400ug/kg, IP). Pink dots indicate

data from females; brown dots indicate data from males. (H-I) Representative images showing CGRP-IR (green) and FOS-IR (red) in the PBN of $Glp1r^{KO}$ (H) and $AP^{Glp1r\text{-}Re}$ (I) mice treated with Sema (10 nmol/kg) for 2 hours. Scale bars=100 um. Insets show digital zooms of the CGRP-IR containing region of each image. One-way ANOVA with Tukey's (G). ns- p>0.05, *p<0.05, **p<0.01, ***p<0.001, ****p<0.0001.

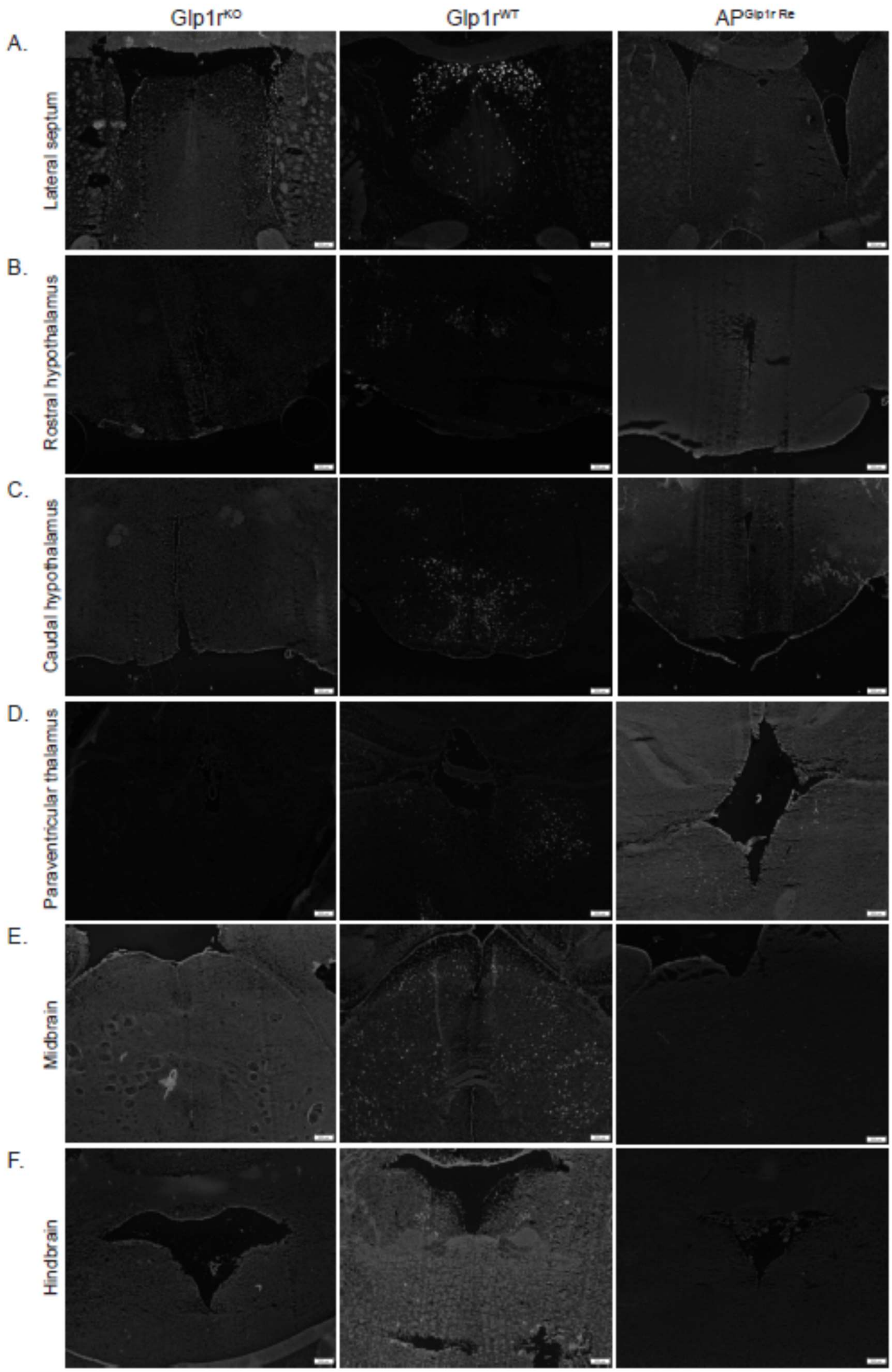
Glp1r^{KO}
Glp1r^{WT}
AP$^{Glp1r\ Re}$
A.
Lateral septum
B.
Rostral hypothalamus
C.
Caudal hypothalamus
D.
Paraventricular thalamus
E.
Midbrain
F.
Hindbrain

Supplemental Figure 6- Brain-wide *Glp1r* ISH.

Glp1r RNA Detection in the whole brain of $Glp1r^{KO}$ (left), $Glp1r^{WT}$ (center), and $AP^{Glp1r\text{-}Re}$ (right) (n=2 each) pseudocolored gray. (A) Lateral septum. (B) Rostral hypothalamus. (C) Caudal Hypothalamus. (D) Paraventricular thalamus. (E) Midbrain. (F) Hindbrain.

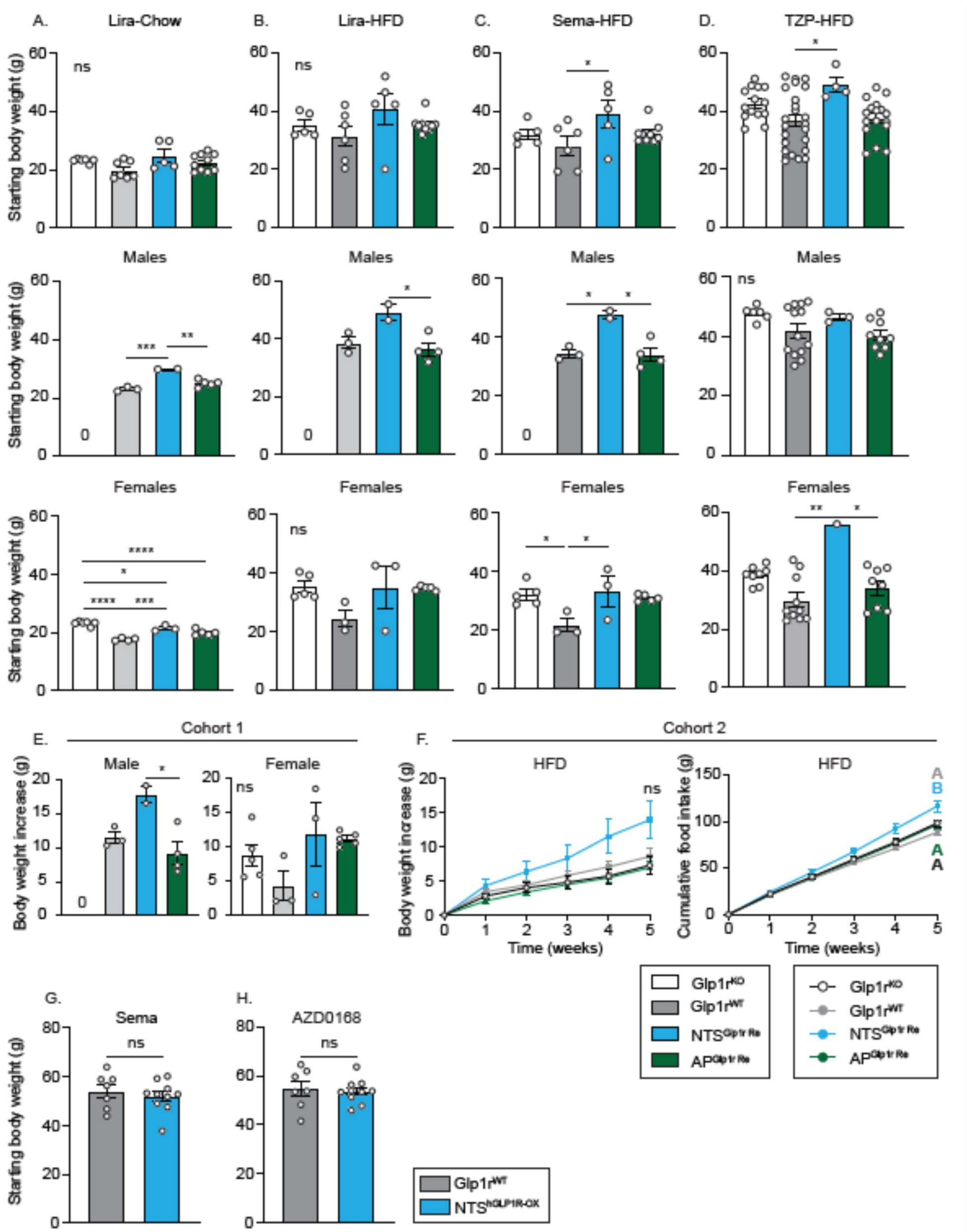

Supplemental Figure 7: Starting body weights for studies in Figures 3-5.
(A) Starting body weight in grams for mice in (Fig 3A-B). (B) Starting body weight in grams for mice in (Fig 3C-F). (C) Starting body weights in grams for mice in (Fig 4A-D).

(D) Starting body weights for mice in (Fig 4E-H). Indicated starting body weights for males (middle) and females (bottom). (E) Change in body weight from HFD-feeding between mice in (A) and (C) broken down by males (left) and females (right). (F) Left, example body weight increase for $Glp1r^{KO}$ (n=14), $Glp1r^{WT}$ (n=23), $AP^{Glp1r\ Re}$(n=17), $NTS^{Glp1r\ Re}$(n=4) on HFD. Right, example cumulative food intake for $Glp1r^{KO}$, $Glp1r^{WT}$, $AP^{Glp1r\ Re}$, $NTS^{Glp1r\ Re}$, and $DVC^{Glp1r\ Re}$ on HFD. Mice represented in (E-F) are the same cochort shown in (D). (G-H) Starting body weights for $Glp1r^{WT}$ (n=7) and $NTS^{hGLP1R\text{-}OX}$ mice (n=10) prior to Sema treatment (Fig 5 E-G) or AZD0186 (Fig 5 H-J). Pink dots indicate data from females; brown dots indicate data from males. One-way ANOVA with Tukey's test for A-E (all), repeated measures two-way ANOVA with Tukey's multiple comparisons for F, unpaired t-test w/Welch's correction for G-H. All graphs: mean +/- SEM shown. NS p>0.05, *p<0.05, **p<0.01, ***p<0.001, ****p<0.0001.

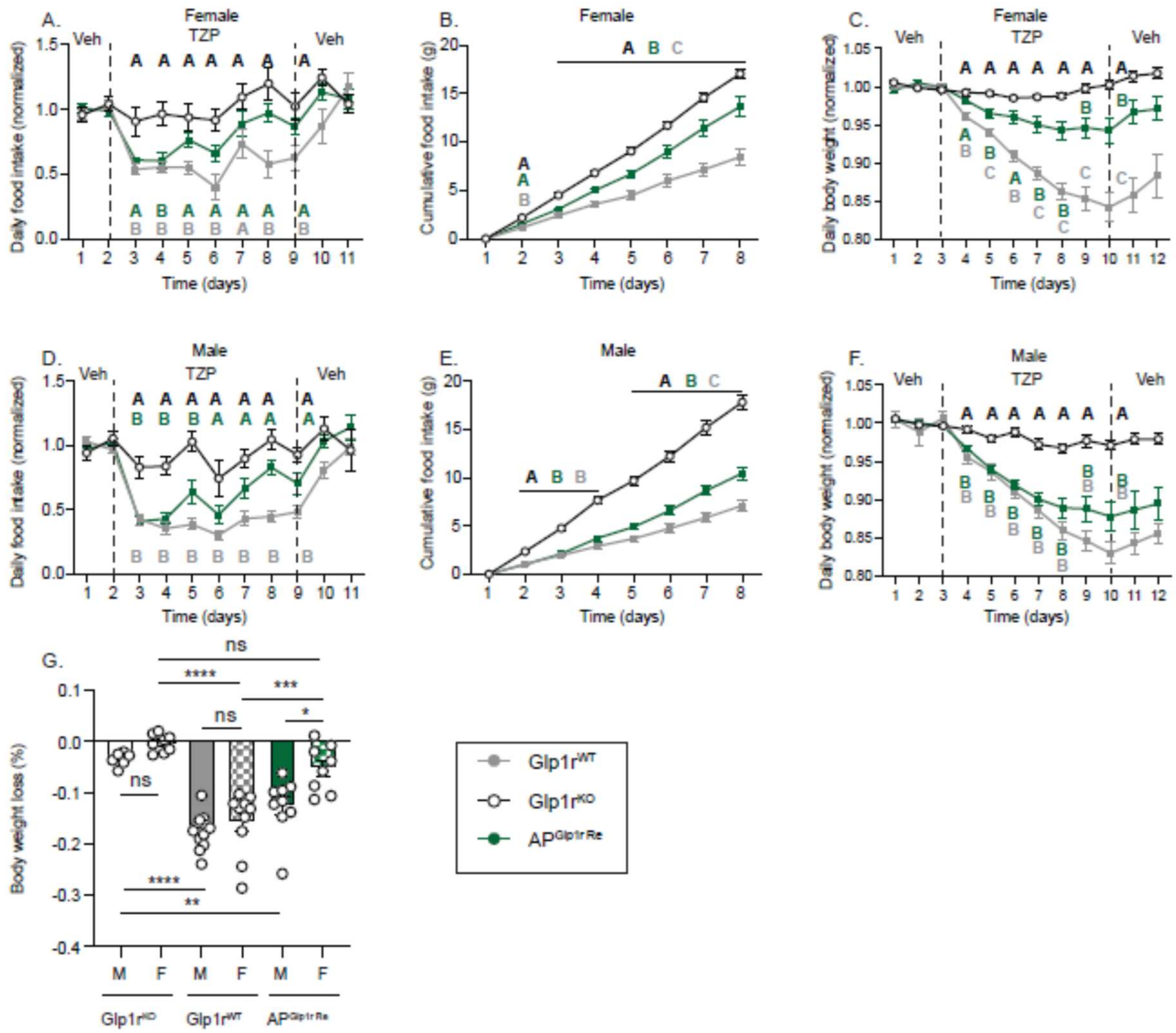

<u>Supplemental Figure 8: Sex specific effects of TZP treatment in AP$^{Glp1r\ Re}$ mice</u>
(A-C) Normalized daily food intake, normalized daily body weight, and cumulative food intake of female Glp1r^{KO} (n=7-8), Glp1r^{WT} (n=10), and AP$^{Glp1r-Re}$ (n=7-8) mice from Fig. 4 E-F) . (D-F) Normalized daily food intake, normalized daily body weight, and cumulative food intake of male Glp1r^{KO} (n=6), Glp1r^{WT} (n=13), and AP$^{Glp1r-Re}$ (n=9) mice from Fig. 4 E-F) . (G) Final body weight loss from Fig 4H broken down by sex. (Glp1r^{KO} (6M/8F), Glp1r^{WT} (13M/10F), and AP$^{Glp1r\ Re}$ (9M/8F)). A-F repeated measures two-way ANOVA with Tukey's multiple comparisons. Two-way ANOVA with Tukey's multiple comparisons for G. All graphs: mean +/- SEM shown. ns- p>0.05, *p<0.05, **p<0.01, ***p<0.001, ****p<0.0001.
